# Vilsmeier reagent, NaHSe and diclofenac acid chloride: one-pot synthesis of a novel selenoindolinone with potent anticancer activity†

**DOI:** 10.1039/d0ra07332f

**Published:** 2020-10-19

**Authors:** Ana Carolina Ruberte, Carlos Aydillo, Arun K. Sharma, Carmen Sanmartín, Daniel Plano

**Affiliations:** Universidad de Navarra, Facultad de Farmacia y Nutrición, Departamento de Tecnología y Química Farmacéuticas Irunla-rrea 1 E-31008 Pamplona Spain sanmartin@unav.es +34-948425600 ext. 806388; Instituto de Investigación Sanitaria de Navarra (IdiSNA) Irunlarrea, 3 31008 Pamplona Spain; Penn State College of Medicine, Penn State Cancer Institute, CH72, Department of Pharmacology 500 University Drive Hershey Pennsylvania 17033 USA

## Abstract

An effective and straightforward synthesis of 3-seleno functionalized indolinone (5) involving Vilsmeier reagent is presented. Likewise, a procedure to achieve lactamization of diclofenac with excellent yields by using hydrides is also ascertained. Compound 5 exhibited impressive growth inhibition in most of the cell lines in an NCI-60 panel, particularly towards resistant breast cancer cells.

## Abstract 2

An effective and straightforward synthesis of 3-seleno functionalized indolinone (5) involving Vilsmeier reagent is presented.

## Graphical abstract

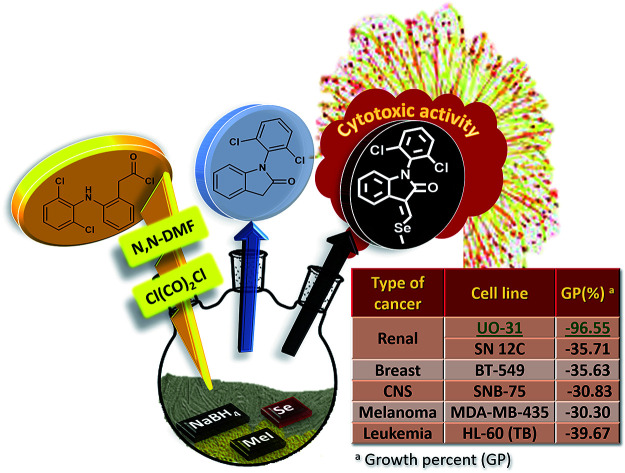

Organoselenium compounds have been broadly studied owing to their well-regarded biological activities.^1–4^ Among the multiple and complex health benefits ascribed to organoselenium compounds, their role as chemotherapy agents is greatly-recognized.^3^ However, their activity is highly dependent on multiple factors, among which metabolic routes stand out. In this context, methylselenol is one of the main metabolites, and has received great attention as a key executor of organoselenium compounds' anticancer activity.^5^

A vast number of molecules containing heterocyclic rings possess a great variety of biological applications, including antitumor activity. Accordingly, indole derivatives show potent antitumoral activity against several cancers, such as lung, pancreatic and breast, in preclinical models.^6–9^ Thus, indole can be considered as a privileged scaffold for designing novel anticancer agents. We hypothesized that the functionalization of indole with a methylseleno group would yield a promising antitumor agent. Therefore, the objective of this work was the synthesis and cytotoxicity evaluation of 1-(2,6-dichlorophenyl)-2-(methylselanyl)-1*H*-indole (compound 4 in Scheme 1). The synthetic procedure designed to obtain the desired derivative is illustrated in Scheme 1.

**Scheme 1:**
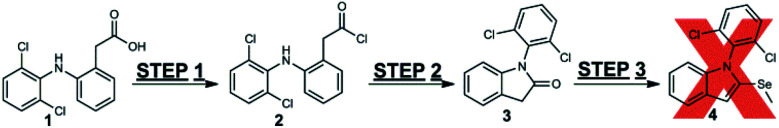
Synthetic route designed for 1-(2,6-dichlorophenyl)-2-(methylselanyl)-1*H*-indole (4).

The designed synthesis was originally planned in three steps. Firstly, the chlorination of diclofenac (1) to achieve the acid chloride 2 (Scheme 1, step 1). Secondly, the intramolecular cyclization of the acid chloride 2 through the nucleophilic attack of the nitrogen over the acyl chloride (Scheme 1, step 2). Finally, a two-steps reaction to yield the desired compound 4*via* formation of the selenoamide and the subsequent methylation of the Se atom (Scheme 1, step 3). Different conditions and reagents have been reported for each of these steps, all of them being optimized. Next, we will discuss some previously reported methods along with our conditions and results for each step.

The chlorination of diclofenac (1) (Scheme 1, step 1) was attempted under three conditions, as previously reported in the literature:^10–12^ (i) reflux conditions with an excess of thionyl chloride (SOCl_2_) for 2 h,^10^ resulting in the degradation of diclofenac (1), observed by ^1^H-NMR spectra; (ii) reaction with oxalyl chloride [Cl(CO)_2_Cl] in methylene chloride (DCM) at room temperature,^11^ that did not yield the acid chloride 2 even at long periods of time (up to 72 h); and (iii) the use of few drops of *N*,*N*-dimethylformamide (DMF) as catalyst for the reaction for oxalyl chloride, which turned out to be the optimal conditions with quantitative yields (99%).^12^

Regarding to molecular cyclization to render the lactam 1-(2,6-dichlorophenyl)indolin-2-one (3) (Scheme 1, step 2), several procedures have been published on the literature: radical photoredox cyclization,^13,14^ acid catalyzed cyclization^15,16^ and Mn(iii)-based oxidative cyclization methods,^17^ as well as 1-ethyl-3-(3-dimethylaminopropyl)carbodiimide (EDC)-based carboxyl activation cyclization,^18^ among others. Nevertheless, these methods present limitations, including multiple steps, complex catalysis and use of expensive reagents. Thus, the intramolecular reductive cyclization based on hydrides (*e.g.* lithium aluminum hydride (LiAlH_4_); lithium tri-*t*-butoxy aluminum hydride (LiAlH(OtBu)_3_);^19^ sodium cyanoborohydride (NaBH_3_CN)^20^ and sodium borohydride (NaBH_4_)^21^) caught our attention because of their straightforward and low cost procedure. Hence, we developed novel and efficient method of intramolecular cyclization with hydrides to obtain lactam 3. This method was achieved by reaction of acid chloride 2 with one equivalent of hydride in water and tetrahydrofuran (THF) at room temperature for 2 h. Besides, we optimized the method using five different hydrides, such as LiAlH_4_, lithium triethylborohydride (LiEt_3_BH), LiAlH(OtBu)_3_, NaBH_3_CN and NaBH_4_ (Table 1). Luckily, we obtained excellent yields with all hydrides, but the better yield was with LiAlH(OtBu)_3_ (Table 1, entry 3).

**Table 1:** The hydride used for cyclization reaction to obtain lactam 3^a^

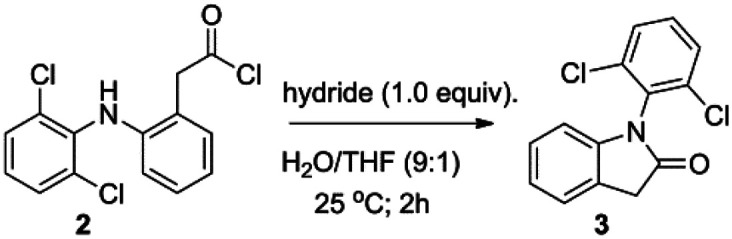		
Entry	Hydride	Yield^b^ (%)
1	AlLiH_4_	46
2	LiEt_3_BH	53
3	LiAlH(OtBu)_3_	82
4	NaBH_3_CN	80
5	NaBH_4_	78

a All reactions were carried out with compound 2 (6.4 mmol) and the corresponding hydride (6.4 mmol) in a mixture of water (18 mL) and THF (2 mL) as solvent at RT for 2 h.

b Estimated yields of 3 determined by ^1^H-NMR.

Finally, we focused on formation of the selenoamide and the subsequent methylation of the Se atom to yield the desired compound, 1-(2,6-dichlorophenyl)-2-(methylselanyl)-1*H*-indole (4) (Scheme 1, step 3). Concerning the selenoamide formation, we considered several reported methods gathered in the literature about the use of hydrogen selenide (H_2_Se) and hydrogen sulfide (H_2_S) in the conversion of amides in thioamides;^22^ thioureas in selenoureas^20^ and imines in selenones,^20^ among others. Thus, amide with H_2_Se should form the selenoamide. However, the toxicity of H_2_Se prompted us to find safe and effective alternative in alkali metal salt of hydroselenide (MHSe), which may be readily prepared *in situ* by the reaction of Se and hydride.^11,23–25^ Another advantage is that hydride was already used in the intramolecular cyclization (Table 1). Hence, the reaction conditions to form the lactam 3 (Scheme 1, step 2) are similar to last step (Scheme 1, step 3), prompting us to perform step 2 and step 3 in one-pot synthesis.

Thus, the formation of desired compound 4 from acid chloride 2 was attempted with oxalyl chloride (0.5 equivalent) and *N*,*N*-dimethylformamide (0.5 equivalent), Se (1.0 equivalent), LiAlH(OtBu)_3_ (2 equivalent to form lactam 3 and subsequently selenamide) and iodomethane (MeI) (3 equivalent) in water and tetrahydrofuran, under two conditions: (i) room temperature, up to 8 days; (ii) reflux, up to 2 days. Unfortunately, these conditions did not yield desired compound 4. In fact, this step did not evolve toward any selenocompound and most of the material recovered was the starting material. This result can be attributed to the lack of formation of lithium hydroselenide (Table S4 and Fig. S12, ESI†) necessary to form selenoamide and subsequently desired compound. Therefore, we studied the formation of MHSe with the rest of hydrides used previously (Table S4 and Fig. S10–S14, ESI†). Remarkably, NaBH_4_ was the only one that formed MHSe (Table S4 and Fig. S14, ESI†).

We decided to repeat the previous methods with NaBH_4_ instead of LiAlH(OtBu)_3_ (Scheme 2). Surprisingly, desired compound 4 was not detected at all, unlike lactam 3 with 49% of yield. Remarkably, we have discovered that the reaction gives rise to an unexpected 3-alkylidene-2-oxindole with Se ((*E*)-1-(2,6-dichlorophenyl)-3-((methylselanyl)methylene)indolin-2-one, 5) with 10% of yield (Scheme 2). This unexpected derivative was purified by column chromatography, characterized by NMR and its structure (Fig. 1) unambiguously determined by X-ray diffraction (CCDC 1983076, Tables S8–S15, ESI†).

**Scheme 2:**
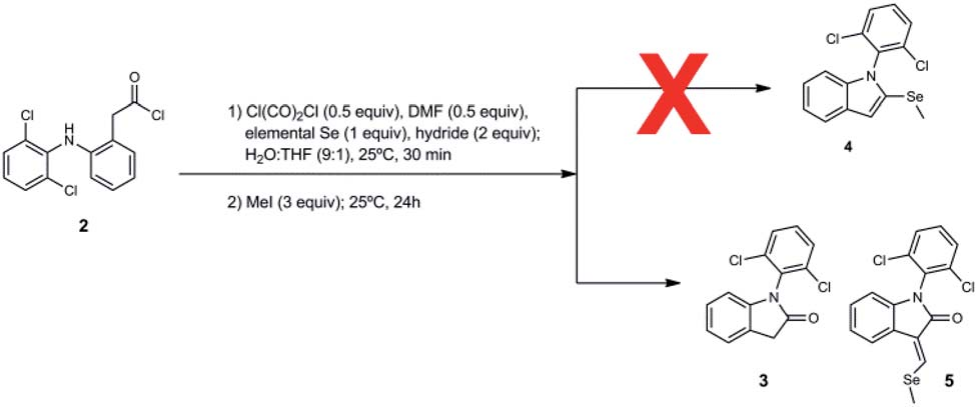
One-pot synthesis of unexpected derivative 5.

**Fig. 1:**
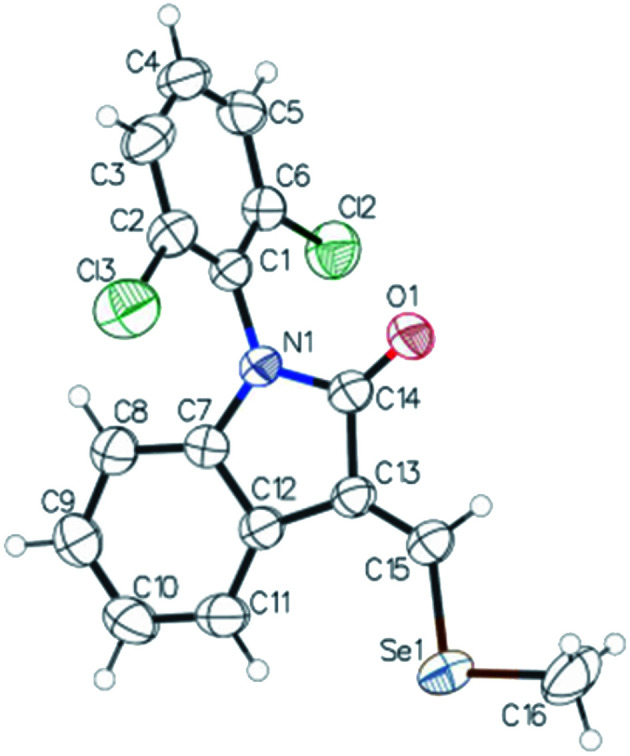
ORTEP diagram of compound 5 with displacement ellipsoids drawn at 50% probability level. Crystal Data for C_16_H_11_Cl_2_NOSe (*M* = 383.12 g mol^−1^): monoclinic, space group *P*2_1_/*c* (no. 14), *a* = 8.3923(9) Å, *b* = 12.7253(14) Å, *c* = 14.6560(15) Å, *β* = 90.072(2)°, *V* = 1565.2(3) Å^3^, *Z* = 4, *T* = 298 K, *μ*(MoKα) = 2.737 mm^−1^, *D*_calc_ = 1.626 g cm^−3^, 13 375 reflections measured (4.24° ≤ 2*Θ* ≤ 56.66°), 3859 unique (*R*_int_ = 0.0227, *R*_sigma_ = 0.0301) which were used in all calculations. The final *R*_1_ was 0.0365 (>2sigma(*I*)) and w*R*_2_ was 0.1049 (all data).

Since isolated diclofenac lactam 3 did not yield selenated product 5 under these conditions, the proposed mechanism (Scheme 3) would need to start from diclofenac carbonyl chloride 2, and involve the formation of selenocarboxylate A by the attack of *in situ* generated hydroselenide (HSe^−^), and posterior esterification with MeI to form **B**. Vilsmeier reagent generated by DMF and oxalyl chloride present in the reaction mixture is attacked by selenomethyl ester enolate, probably mediated by the presence of NaBH_4_, generating after hydrolysis the formylated intermediate C. Examples of related formylation of esters with alkyl formats in presence of base (hydrides^26^ and alkoxides^27^) have been previously reported. The next step would involve the intramolecular amide formation by the attack of secondary amine to the activated selenomethyl ester, generating intermediate D. Leaving methyl selenide can attack nucleophilically at aldehyde carbon, generating E, which could easily undergo water elimination, taking into account the additional stabilization gained by conjugation with aromatic system and the lactam in the final product 5. Low yields of product 5 are justified as lactamization is a favored process, even more with activated species, like acid chlorides and selenoesters, which have been known for acyl activation in peptide synthesis^28^ and protein chemical ligation.^29^

**Scheme 3:**
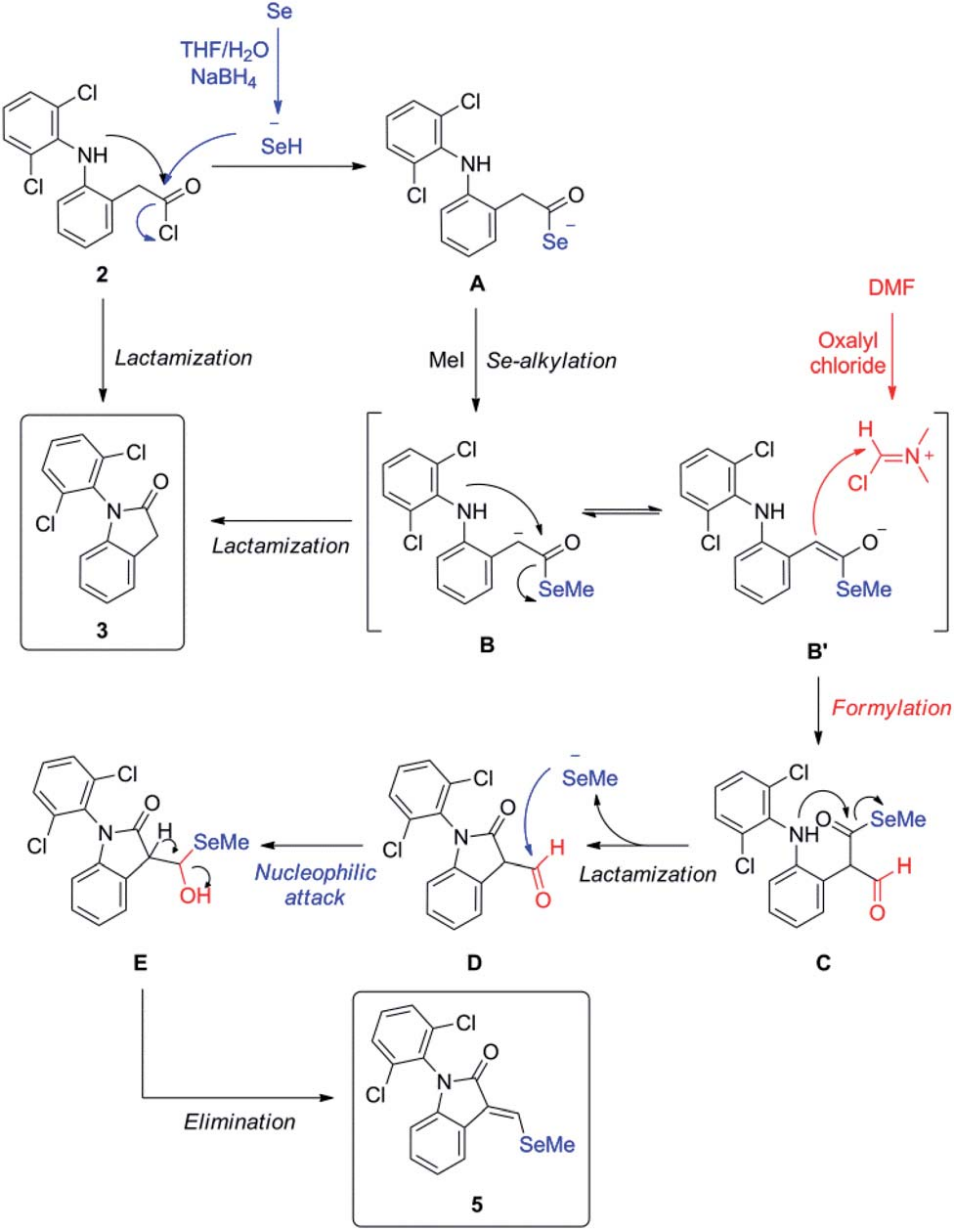
Proposed reaction mechanism one-pot synthesis of unexpected derivative 5.

Once that Vilsmeier reagent is suggested to be a key part of product 5 generation, we attempted to optimize the method using more equivalents of DMF than in previously used method (Table 2). Nevertheless, the yield of unexpected derivative 5 and lactam 3 decreased probably due to the increased solubility of the organic compounds in the reaction medium and the subsequent additional issues to isolate the desired compounds.

**Table 2:** Optimization of reaction conditions of unexpected derivative 5^a^

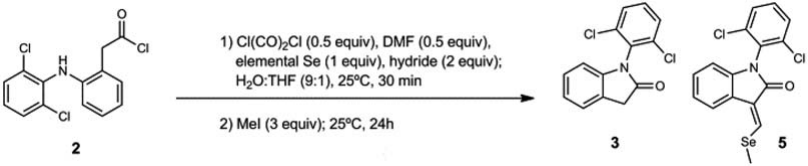				
Entry	DMF (equiv.)	Cl(CO)_2_Cl (equiv.)	Yield (%) comp. 3^b^	Yield (%) comp. 5^b^
1	0	0	78	NR^c^
2	0.5	0.5	58	14
3	1	0.5	49	2
4	1.5	0.5	47	1<

a All reactions were carried out with compound 2 (6.4 mmol), oxalyl chloride (3.2 mmol) and DMF (0, 3.2, 6.4 or 9.6 mmol), elemental selenium (6.4 mmol) and NaBH_4_ (12.8 mmol) in a mixture of water and THF (9 : 1) at RT. After 30 min, MeI (19.2 mmol) was added to the reaction and stirred at RT for 24 h.

b Estimated yields of 3 and 5 determined by ^1^H-NMR.

c No reaction.

Finally, this unexpected new organoselenium derivative 5 with the code NSC: 811012 was submitted to the National Cancer Institute's (NCI) Developmental Therapeutics Program (DTP), to be tested at a single dose of 10 μM in a panel of NCI-60 human tumor cell lines. These cell lines include nine cancer types (leukemia, melanoma, non-small cell lung, colon, CNS, ovarian, renal, prostate and breast cancers).^30^ The results (Fig. S1, ESI†) were expressed in growth percent (GP), that is the growth of treated cells compared to the growth of an untreated cells. GP (%) values between 0 and 50 means antiproliferative properties and between −100 and 0 stand for cytotoxic properties. Compound 5 demonstrated antitumor activity in most NCI-60 cell lines, with a GP mean value towards all cell lines of 17.48% (Fig. S1, ESI†). In addition, compound 5 showed potent cytotoxic activity with GP lower than −30.80% in 6 out of the 60 tumor cell lines tested, including renal (UO-31 and SN12C), breast (BT-549), CNS (SNB-75), melanoma (MDA-MB-435) and leukemia (HL-60 (TB)) cancers (Fig. 2). In this context, compound 5 exhibited the most cytotoxic activity (GP equal to −96.55%) towards UO-31, a renal cancer cell line. Additionally, this new derivative showed great cytotoxic activity (GP equal to −35.63%) in the most resistant breast cancer cell of NCI panel towards more than 20 000 compounds tested (BT-549).^30^

**Fig. 2:**
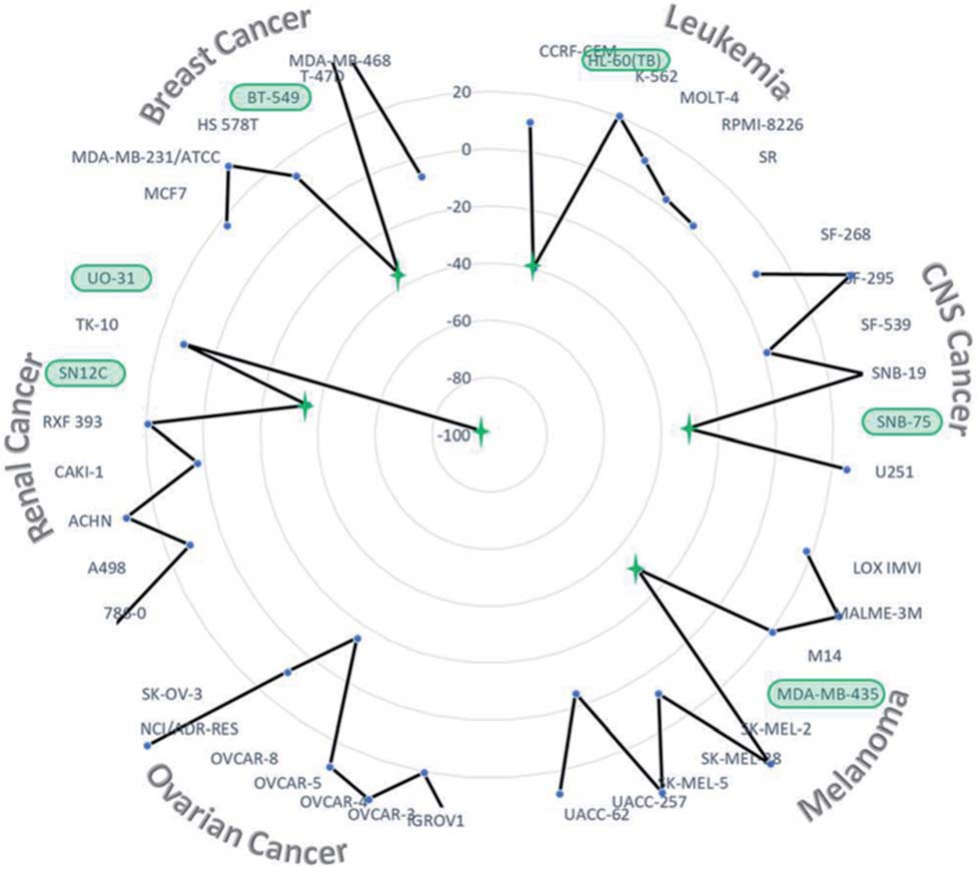
Growth percent values towards several breast, renal, ovarian, melanoma, CNS and leukaemia cancer cell lines.

In conclusion, we developed a novel and efficient method of intramolecular cyclization with hydrides to obtain lactam 3. Besides, we report an unexpected and unprecedented Vilsmeier reagent application to afford a selenated lactam 5 with potent cytotoxic activity in resistant breast cancer cells. Hence, this new synthesis may be an attractive approach for the discovery of new potent antitumoral agents, particularly for therapy of resistant breast cancers.

## Conflicts of interest

There are no conflicts to declare.

## Supplementary Material

RA-010-D0RA07332F-s001

RA-010-D0RA07332F-s002
