# Supplementary material for: Vilsmeier reagent, NaHSe and diclofenac acid chloride: one-pot synthesis of a novel selenoindolinone with potent anticancer activity
Source: RSC Adv. 2020 Oct 19;10(63):38404–8. doi: 10.1039/d0ra07332f (PMC9057275; doi:10.1039/d0ra07332f)
Supplement: RA-010-D0RA07332F-s001 [file RA-010-D0RA07332F-s001.pdf]

Electronic Supplementary Material (ESI) for RSC Advances.
This journal is © The Royal Society of Chemistry 2020

Vilsmeier reagent, NaHSe and diclofenac acid chloride: one-pot synthesis of a novel selenoindolinone with potent anticancer activity

Ana Carolina Ruberte,[a] Carlos Aydillo,[a] Arun K. Sharma,[b] Carmen Sanmartín, *[a] Daniel Plano[a]

[a] Universidad de Navarra, Facultad de Farmacia y Nutrición, Departamento de Tecnología y Química Farmacéuticas, Irunlarrea 1, E-31008 Pamplona, Spain. Instituto de Investigación Sanitaria de Navarra (IdiSNA), Irunlarrea, 3, 31008 Pamplona, Spain

[b] Penn State College of Medicine, Penn State Cancer Institute, CH72, Department of Pharmacology, 500 University Drive, Hershey, Pennsylvania 17033, United States

*Correspondence: sanmartin@unav.es; Tel.: +34-948425600 ext. 806388

Table of Contents

I. General information (**Table S1**)
II. Methods
II.1. Synthetic procedure
II.1.1. 2-(2-((2,6-dichlorophenyl)amino)phenyl)acetyl chloride (**2**)
II.1.2. 1-(2,6-dichlorophenyl)indolin-2-one (**3**)
II.1.3. Alkali metal salt of hydroselenide
II.1.4. 1-(2,6-dichlorophenyl)-2-(methylselanyl)-1*H*-indole (**4**)
II.1.5. Reaction optimization of ((*E*)-1-(2,6-dichlorophenyl)-3-((methylselanyl)methylene)indolin-2-one (**5**)
II.2. Quantitative NMR (qNMR)
*II.3. X-ray diffractometry procedure for compound **5***
*II.4. Biological evaluation for compound **5***
III. Results
III.1. Synthesis
III.1.1. 2-(2-((2,6-dichlorophenyl)amino)phenyl)acetyl chloride (**1**) (**Table S2**)
III.1.2. 1-(2,6-dichlorophenyl)indolin-2-one (**3**) (**Table S3**)
III.1.3. Alkali metal salt of hydroselenide (**Table S4**)
III.1.4. ((*E*)-1-(2,6-dichlorophenyl)-3-((methylselanyl)methylene)indolin-2-one (**5**) (**Table S5**)
III.1.5. Reaction optimization of ((*E*)-1-(2,6-dichlorophenyl)-3-((methylselanyl)methylene)indolin-2-one (**5**) (**Table S6**)
III.1.6. Optimization of reagent addition sequence for derivative **5** (**Table S7**)
*III.2. X-ray diffractometry data for compound **5*** (**Tables S8-15**)
*III.3. Biological evaluation for compound **5***
III.3.1. NCI-60 screening data for compound **5** (**Figure S1**)
IV. Spectroscopic characterization for compound 3 and 5.
*IV.1. 1-(2,6-dichlorophenyl)indolin-2-one (**3**)*
*IV.2. ((E)-1-(2,6-dichlorophenyl)-3-((methylselanyl)methylene)indolin-2-one (**5**)*
IV.3. NMR spectra and quantitative NMR (qNMR) **(Figures S2-S7)**
V. ^{1}H-NMR spectra for reaction in the synthesis of 2-(2-((2,6-dichlorophenyl)amino)phenyl)acetyl chloride (2) (Figures S8-S9).
VI. ^{77}Se-NMR spectra for reaction crudes in the synthesis of alkali metal salts of hydroselenide (Figures S10-S14)
VII. ^{1}H-NMR spectra with dimethylsulfone for quantification of crude mixture
*VII.1. 1-(2,6-dichlorophenyl)indolin-2-one (**3**)* (**Figures S15-S19**)
VII.2. Optimization of cyclization with N, N- dimethylformamide (**Figures S20-S22**)

I. General information

^{1}H NMR and ^{13}C NMR spectra were measured with tetramethylsilane as an internal standard, on a Bruker Avance 600 and Bruker Avance Neo 400 instruments in CDCl3, operating at 400, 500 or 600, and 100, 125 or 150 MHz, respectively. Chemical shifts are reported in δ values (ppm) and coupling constants (*J*) values are reported in Hz. ^{77}Se NMR spectra were recorded on a Bruker Avance Neo 400 operating at 76 MHz, using Me_2Se_2 as external reference. Melting points were taken with a micro melting point apparatus. The most of starting materials and solvents were purchased from commercial suppliers and were used as received. Reaction courses were monitored by thin-layer chromatography (TLC) on precoated silica gel 60 F254 aluminum sheets (Merck, Darmstadt, Germany). The crude reaction product was purified by silica gel column chromatography using silica gel 60 Å (Merck, 230–400 mesh), and hexane/ ethyl acetate (Table S1) was used as the elution solvent.

Table S1. Eluent ratios used for chromatographic column purification of compounds **3** and **5**, both obtained from the same reaction crude, CV meaning column volume.

Mix Solvent	Rf values [hexane : ethyl acetate; 8 : 2)]
3 CV of hexane	0.89
3 CV of hexane : ethyl acetate (95 : 5)	0.53
3 CV of hexane : ethyl acetate (93 : 7)	0.41 (Compound.**3**)
4 CV to hexane : ethyl acetate (92 : 8)	0.35 (Compound. **5**)

II. Methods

II.1. Synthetic procedure

II.1.1. 2-(2-((2,6-dichlorophenyl)amino)phenyl)acetyl chloride (**2**)

Diclofenac sodium was dissolved in distilled water until a homogeneous solution was obtained. Then, excess of concentrated hydrochloric acid was added to obtain pure diclofenac acid as a precipitate [1].

Procedure A: The chlorination of diclofenac (**1**) was attempted by reaction of the previously obtained diclofenac acid (2 g, 8.8 mmol) with an excess of thionyl chloride (6.40 mL, 88 mmol), under reflux for 2h [2]. The resulting acyl chloride was isolated by rotatory evaporation of the thionyl chloride under reduce pressure and the excess of thionyl chloride was removed with 3 fractions of toluene (3 x 40 mL).

Procedure B: The chlorination of diclofenac (**1**) was attempted by reaction of the previously obtained diclofenac acid (2 g, 8.8 mmol) in methylene chloride (DCM) (20 mL) with oxalyl chloride (2.34 mL, 26.4 mmol) at room temperature (RT) for 2 to 72 h [3]. The resulting acyl chloride was isolated by rotatory evaporation of the DCM under reduce pressure.

Procedure C: The chlorination of diclofenac (**1**) was achieved by reaction of the previously obtained diclofenac acid (2 g, 8.8 mmol) in DCM (20 mL) with oxalyl chloride (2.34 mL, 26.4 mmol) and *N*, *N*-dimethylformamide (0.34 mL, 4.4 mmol) at RT for 2h [3]. The resulting acyl chloride was isolated by rotatory evaporation of the DCM under reduce pressure.

II.1.2. 1-(2,6-dichlorophenyl)indolin-2-one (**3**)

The reaction was performed with 2-(2-((2,6-dichlorophenyl)amino)phenyl)acetyl chloride (**2**) (2 g, 6.4 mmol) and LiAlH(OtBu)$_3$ (1.6 g, 6.4 mmol), $NaBH_3CN$ (0.4 g, 6.4 mmol), $NaBH_4$ (0.3 g, 6.4 mmol), $LiEt_3BH$ (0.7 g, 6.4 mmol) or $LiAlH_4$ (0.2 g, 6.4 mmol), in a mixture of water (18 mL) and tetrahydrofuran (2 mL) as solvent at RT for 2h. Then, the reactions mixtures were extracted with DCM (3 x 20 mL). The organic layers were dried with magnesium sulfate and concentrated under reduce pressure.

II.1.3. Alkali metal salt of hydroselenide

The reaction was performed with elemental selenium (0.5g, 6.4 mmol) and the corresponding hydride previously used [LiAlH(OtBu)$_3$ (1.6 g, 6.4 mmol), $NaBH_3CN$ (0.4 g, 6.4 mmol), $NaBH_4$ (0.3 g, 6.4 mmol), $LiEt_3BH$ (0.7 g, 6.4 mmol) or $LiAlH_4$ (0.2 g, 6.4 mmol)] in water (5 mL) as solvent at RT for 10 min.

II.1.4. 1-(2,6-dichlorophenyl)-2-(methylselanyl)-1*H*-indole (**4**)

<u>Procedure A:</u> The reaction was carried out using derivative **2** (2 g, 6.4 mmol), oxalyl chloride (0.28 mL, 3.2 mmol) and *N*, *N*-dimethylformamide (0.25 mL, 3.2 mmol), elemental selenium (0.5g, 6.4 mmol) and LiAlH(OtBu)$_3$ (3.2 g, 12.8 mmol) in a mixture of water and tetrahydrofuran (9: 1) at RT for 2 h. Then, iodomethane (1.2 mL, 19.2 mmol) was added to the mixture and the reaction was stirred at RT for 8 days.

<u>Procedure B:</u> The reaction was carried out using derivative **2** (2 g, 6.4 mmol), oxalyl chloride (0.28 mL, 3.2 mmol) and *N*, *N*-dimethylformamide (0.25 mL, 3.2 mmol), elemental selenium (0.5g, 6.4 mmol) and LiAlH(OtBu)$_3$ (3.2 g, 12.8 mmol) in a mixture of water and tetrahydrofuran (9: 1) at RT for 2 h. Then, iodomethane (1.2 mL, 19.2 mmol) was added to the mixture and the reaction was stirred under reflux for 2 hours.

<u>Procedure C:</u> The reaction was carried out using derivative **2** (2 g, 6.4 mmol), oxalyl chloride (0.28 mL, 3.2 mmol) and *N*, *N*-dimethylformamide (0.25 mL, 3.2 mmol), elemental selenium (0.5 g, 6.4 mmol) and $NaBH_4$ (0.5 g, 12.8 mmol) in a mixture of water and tetrahydrofuran (9: 1) at RT for 2 h. Then, iodomethane (1.2 mL, 19.2 mmol) was added to the mixture and the reaction was stirred at RT for 24 h.

The reaction mixture was extracted with methylene chloride (3 x 20 mL). The organic layers were combined and dried over magnesium sulfate and concentrated under reduced pressure.

II.1.5. Reaction optimization of ((*E*)-1-(2,6-dichlorophenyl)-3-((methylselanyl)methylene)indolin-2-one (**5**)

The chlorination of diclofenac was optimized by reaction of diclofenac acid (2 g, 8.8 mmol) in DCM (20 mL) with oxalyl chloride (2.34 mL, 26.4 mmol) and *N*, *N*-dimethylformamide (4.4 mmol) at RT for 2h. The resulting compound **2** was isolated by rotatory evaporation under vacuum. Then, compound **2** (2 g, 6.4 mmol), oxalyl chloride (0.28 mL, 3.2 mmol) and *N*, *N*-dimethylformamide (3.2, 6.4 or 9.6 mmol), elemental selenium (0.5 g, 6.4 mmol) and $NaBH_4$ (0.5 g, 12.8 mmol) were mixed in water and tetrahydrofuran (9: 1) at RT. After 30 min, iodomethane (1.2 mL, 19.2 mmol) was added to the reaction and stirred at RT for 24 h. Finally, reaction mixture was extracted

with DCM (3 x 20 mL) and the organic layers were combined and dried over magnesium sulfate and concentrated under reduced pressure.

II.2. Quantitative NMR (qNMR)

Quantitative NMR (qNMR) were registered on a Bruker Avance Neo 400 spectrometer using dimethyl sulfone (SigmaAldrich, Ref. #: 41867, CAS #: 67-71-0) as standard to determine the purity of compound 5 [4, 5].

*II.3. X-ray diffractometry procedure for compound **5***

Single crystals of ((*E*)-1-(2,6-dichlorophenyl)-3-((methylselanyl)methylene)indolin-2-one (**5**), were grew from the solution of hexane. A suitable crystal was selected and mounted on a 'CCD area detector' diffractometer (Bruker SMART-APEX), using a nylon loop. The crystal was at 298 K during data collection. Using Olex2 [6], the structure was solved with the XS structure solution program using Direct Methods and refined with the XL [7] refinement package using Least Squares minimization.

*II.4. Biological evaluation for compound **5***

Compound **5** was submitted to the National Cancer Institute's (NCI) Developmental Therapeutics Program (DTP) where its cytotoxicity was screened towards a panel of 60 human cancer cell lines, at one dose and 48 h of treatment [8-11]. Briefly, cells were seeded in 96 well plates and incubated for 24 h. Then, some of the plates were processed to determine the zero time density, and compound **5** were added at 10µM on the remaining plates. Plates were incubated 48 h with the treatment and then fixed and stained with sulforhodamine B. Growth inhibition is calculated relative to cells without drug treatment and the zero time control.

III. Results

III.1. Synthesis

III.1.1. 2-(2-((2,6-dichlorophenyl)amino)phenyl)acetyl chloride (**2**)

Table S2. Synthesis conditions to obtain 2-(2-((2,6-dichlorophenyl)amino)phenyl)acetyl chloride (**2**).

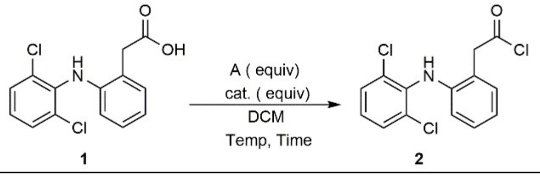

1 2

Entry	A (equiv)	cat. (equiv)	Temp (° C)	Time (min)	Yield (%) [a]
1	$Cl(CO)_2Cl$ (3.0)	---	25	2 to 72 h	NR[b]
2	$Cl(CO)_2Cl$ (3.0)	DMF (0.5)	25	2 h	100
3	$SOCl_2$ (10.0)	---	150	2 h	Trace

[a] Estimated yield of **2** determined by ^{1}H-NMR.

[b] No reaction

III.1.2. 1-(2,6-dichlorophenyl)indolin-2-one (**3**)

Table S3. The hydride used for cyclization reaction to obtain 1-(2,6-dichlorophenyl)indolin-2-one (**3**).

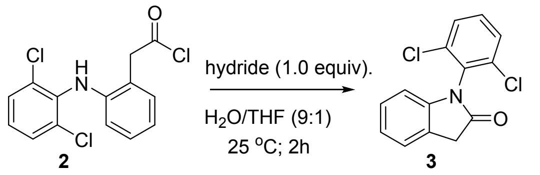

Entry	Hydride	Yield (%) [a]
1	$AlLiH_4$	46
2	$LiEt_3BH$	53
3	$LiAlH(OtBu)_3$	82
4	$NaBH_3CN$	80
5	$NaBH_4$	78

[a] Estimated yields of **3** determined by ^{1}H-NMR.

III.1.3. Alkali metal salts of hydroselenide

Table S4. Synthesis of alkali metal salts of hydroselenide.

$$\text{Se (1.0 equiv)} + \text{MH (1.0 equiv)} \xrightarrow[25\ ^{o}\text{C; 10 min}]{H_2O} M^+HSe^-$$

Entry	MH	Yield (%) [a]
1	$LiAlH_4$	NR[b]
2	$LiEt_3BH$	NR
3	$LiAlH(OtBu)_3$	NR
4	$NaBH_3CN$	NR
5	$NaBH_4$	100

[a] Estimated yield of MHSe determined by ^{1}H-NMR.

[b] No reaction

III.1.4. ((*E*)-1-(2,6-dichlorophenyl)-3-((methylselanyl)methylene)indolin-2-one (**5**)

Table S5. Synthetic scheme and conditions for derivative **5**.

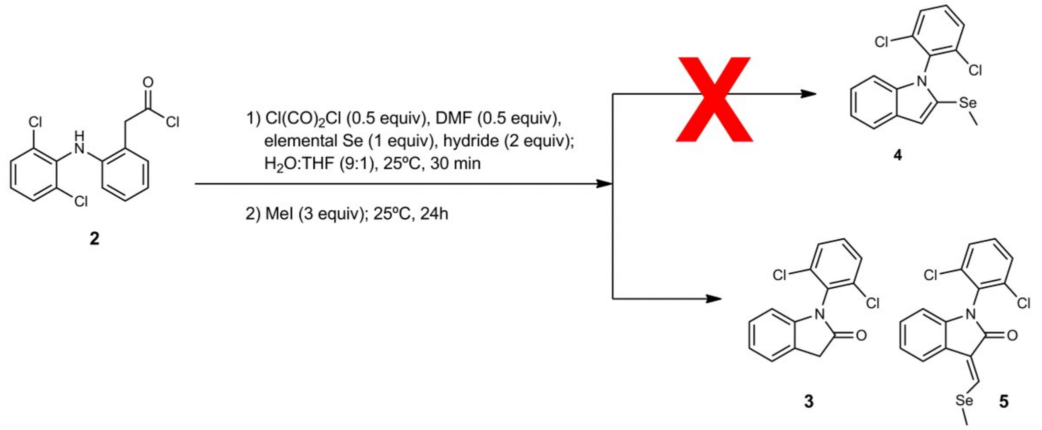

Entry	Hydride	Temp (° C)	Time (days)	Yield (%) comp. **3** [a]	Yield (%) comp. **4** [a]	Yield (%) comp. **5** [a]
1	$LiAlH(OtBu)_3$	25	8	NR [b]	NR	NR
2	$LiAlH(OtBu)_3$	Reflux	2	NR	NR	NR
3	$NaBH_4$	25	1	58	NR	14

[a] Estimated yields of **3**, **4** and **5** determined by ^{1}H-NMR.

[b] No reaction

III.1.5. Reaction optimization of ((*E*)-1-(2,6-dichlorophenyl)-3-((methylselanyl)methylene)indolin-2-one (**5**)

Table S6. Optimization of reaction conditions for derivative **5**.

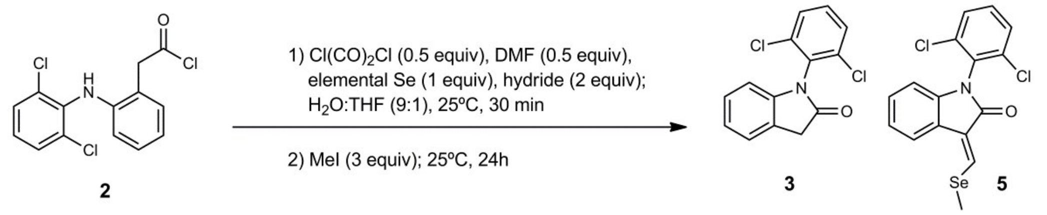

Entry	DMF (equiv)	Yield (%) comp. **3** [a]	Yield (%) comp. **5** [a]
1	0	78	NR[b]
2	0.5	58	14
3	1	49	2
4	1.5	47	1<

[a] Estimated yields of **3** and **5** determined by ^{1}H-NMR.

[b] No reaction

III.1.6. Optimization of reagent addition sequence for derivative **5**

Table S7. Optimization of reagent addition sequence for derivative **5**.

**Order of addition for the reagents after formation of NaHSe**			
Comp. 2	MeI	Vilsmeier reagent	Yield (%)
1	1	1	6[a]
1	2	2	10

[a] Estimated yields for compound **5** determined by ^{1}H-NMR.

*III.2. X-ray diffractometry data for compound **5** (CCDC 1983076)*

Table S8. Crystal data and structure refinement.

**Identification code**	aks14 (Comp. **5**)
**Empirical formula**	$C_{16}H_{11}Cl_2NOSe$
**Formula weight**	383.12
**Temperature/K**	298
**Crystal system**	monoclinic
**Space group**	$P2_1/c$
**a/Å**	8.3923(9)
**b/Å**	12.7253(14)
**c/Å**	14.6560(15)
**α/°**	90.00
**β/°**	90.072(2)
**γ/°**	90.00
**Volume/Å^3**	1565.2(3)
**Z**	4
**ρ_{calc}g/cm^3**	1.626
**μ/mm^{-1}**	2.737
**F(000)**	760.0
**Crystal size/mm^3**	0.21 × 0.15 × 0.11
**Radiation**	MoKα (λ = 0.71073)
**2Θ range for data collection/°**	4.24 to 56.66
**Index ranges**	$-10 \le h \le 11, -16 \le k \le 16, -19 \le l \le 19$
**Reflections collected**	13375
**Independent reflections**	3859 [R_{int} = 0.0227, R_{sigma} = 0.0301]
**Data/restraints/parameters**	3859/0/191
**Goodness-of-fit on F^2**	1.037
**Final R indexes [I>=2σ (I)]**	R_1 = 0.0365, wR_2 = 0.0966

**Final R indexes [all data]**	R_1 = 0.0531, wR_2 = 0.1049
**Largest diff. peak/hole / e Å^{-3}**	0.50/-0.24

Table S9. Fractional Atomic Coordinates (×104) and Equivalent Isotropic Displacement Parameters (Å2×103) for compound **5**. Ueq is defined as 1/3 of of the trace of the orthogonalised UIJ tensor.

**Atom**	*x*	*y*	*z*	U(eq)
**Se1**	1006.7(3)	5009.3(2)	6621.68(16)	53.51(12)
**Cl2**	2002.5(9)	3902.2(5)	2002.0(5)	63.5(2)
**Cl3**	4419.8(10)	895.6(7)	4197.9(5)	75.8(2)
**O1**	4211(2)	4226.3(14)	4013.9(12)	58.9(5)
**N1**	2510(2)	2825.8(15)	3800.9(12)	42.7(4)
**C1**	3345(2)	2389.7(18)	3045.4(14)	40.6(5)
**C2**	4319(3)	1525(2)	3147.0(16)	48.1(5)
**C3**	5210(3)	1137(2)	2428.3(18)	58.0(7)
**C4**	5147(3)	1634(2)	1596.7(17)	56.9(7)
**C5**	4182(3)	2493(2)	1469.8(16)	52.0(6)
**C6**	3273(3)	2856.1(18)	2190.8(15)	42.7(5)
**C7**	1144(3)	2417.1(18)	4230.8(14)	42.1(5)
**C8**	283(3)	1534(2)	4010.9(19)	60.4(7)
**C9**	-1031(4)	1304(2)	4552(2)	70.6(8)
**C10**	-1455(3)	1938(2)	5270(2)	66.9(8)
**C11**	-580(3)	2818(2)	5488.4(17)	54.2(6)
**C12**	749(3)	3069.4(18)	4972.8(14)	40.2(5)
**C13**	1920(3)	3908.9(17)	5009.0(13)	38.4(5)
**C14**	3039(3)	3729.0(18)	4239.3(14)	42.6(5)
**C15**	2191(3)	4699.4(19)	5594.0(14)	42.0(5)
**C16**	2231(4)	6197(3)	7057(2)	81.1(10)

Table S10. Anisotropic Displacement Parameters (Å2×103) for compound **5**. The Anisotropic displacement factor exponent takes the form: -2π2[h2a*2U11+2hka*b*U12+…].

**Atom**	U_{11}	U_{22}	U_{33}	U_{23}	U_{13}	U_{12}
**Se1**	57.03(19)	61.8(2)	41.73(15)	-12.05(10)	4.76(11)	6.51(11)
**Cl2**	69.0(4)	54.5(4)	66.8(4)	5.2(3)	-0.7(3)	9.3(3)
**Cl3**	92.9(6)	78.4(5)	56.3(4)	11.9(3)	-3.1(4)	20.9(4)
**O1**	63.5(11)	57.9(11)	55.3(10)	-12.7(8)	17.2(8)	-23.3(9)
**N1**	46.4(11)	41.1(10)	40.8(9)	-10.4(7)	9.5(8)	-6.9(8)
**C1**	40.6(12)	41.0(12)	40.2(11)	-8.6(9)	6.0(9)	-5.1(9)
**C2**	52.1(13)	49.2(14)	42.8(11)	-6.2(10)	0.0(10)	1.2(11)
**C3**	54.8(15)	54.7(16)	64.7(16)	-13.9(12)	5.8(12)	12.0(12)
**C4**	54.6(15)	63.9(17)	52.2(14)	-18.3(12)	14.8(11)	-3.2(13)
**C5**	58.6(15)	56.5(15)	40.8(11)	-4.6(10)	8.5(10)	-11.3(12)
**C6**	43.1(12)	40.6(12)	44.3(11)	-3.7(9)	3.6(9)	-3.7(9)
**C7**	42.1(12)	41.5(12)	42.6(11)	-2.2(9)	6.3(9)	-2.3(10)
**C8**	59.2(16)	56.2(16)	65.7(16)	-18.7(12)	14.4(13)	-13.8(13)
**C9**	62.1(18)	63.9(19)	86(2)	-17.4(15)	19.4(15)	-23.2(14)
**C10**	54.4(16)	77(2)	69.4(17)	-8.3(15)	20.4(13)	-19.9(14)
**C11**	50.6(14)	62.4(17)	49.5(13)	-6.7(11)	12.7(11)	-2.1(12)
**C12**	42.1(12)	40.2(12)	38.2(10)	-1.5(9)	1.8(9)	2.7(10)
**C13**	43.1(12)	39.1(12)	32.9(10)	-1.4(8)	1.4(8)	2.5(9)
**C14**	47.4(13)	43.7(13)	36.6(10)	-5.2(9)	3.9(9)	-4.1(10)
**C15**	46.1(13)	42.7(12)	37.1(11)	-2.6(9)	-1.2(9)	4.5(10)
**C16**	74(2)	85(2)	84(2)	-48.0(18)	1.1(16)	-5.7(16)

Table S11. Bond Lengths for compound **5**.

**Atom**	Atom	Length/Å	Atom	Atom	Length/Å
**Se1**	C15	1.848(2)	C4	C5	1.373(4)
**Se1**	C16	1.935(3)	C5	C6	1.384(3)
**Cl2**	C6	1.728(2)	C7	C8	1.375(3)
**Cl3**	C2	1.738(3)	C7	C12	1.408(3)
**O1**	C14	1.216(3)	C8	C9	1.389(4)
**N1**	C1	1.424(3)	C9	C10	1.373(4)
**N1**	C7	1.408(3)	C10	C11	1.377(4)
**N1**	C14	1.389(3)	C11	C12	1.385(3)
**C1**	C2	1.379(3)	C12	C13	1.453(3)
**C1**	C6	1.387(3)	C13	C14	1.487(3)
**C2**	C3	1.383(3)	C13	C15	1.341(3)
**C3**	C4	1.374(4)			

Table S12. Bond Angles for compound **5**.

**Atom**	Atom	Atom	Angle/˚	Atom	Atom	Atom	Angle/˚
**C15**	Se1	C16	98.62(12)	C8	C7	N1	128.7(2)
**C7**	N1	C1	127.27(18)	C8	C7	C12	122.6(2)
**C14**	N1	C1	121.66(18)	C7	C8	C9	117.1(2)
**C14**	N1	C7	111.01(17)	C10	C9	C8	121.4(3)
**C2**	C1	N1	121.3(2)	C9	C10	C11	121.1(2)
**C2**	C1	C6	117.6(2)	C10	C11	C12	119.4(2)
**C6**	C1	N1	121.0(2)	C7	C12	C13	107.54(18)
**C1**	C2	Cl3	119.44(18)	C11	C12	C7	118.4(2)
**C1**	C2	C3	121.6(2)	C11	C12	C13	134.0(2)
**C3**	C2	Cl3	119.0(2)	C12	C13	C14	106.69(18)
**C4**	C3	C2	119.4(2)	C15	C13	C12	133.6(2)
**C5**	C4	C3	120.6(2)	C15	C13	C14	119.6(2)
**C4**	C5	C6	119.2(2)	O1	C14	N1	124.3(2)
**C1**	C6	Cl2	120.03(17)	O1	C14	C13	129.7(2)
**C5**	C6	Cl2	118.40(18)	N1	C14	C13	106.05(18)
**C5**	C6	C1	121.6(2)	C13	C15	Se1	126.18(19)
**N1**	C7	C12	108.71(19)				

Table S13. Hydrogen Bonds for compound **5.**

**D**	H	A	d(D-H)/Å	d(H-A)/Å	d(D-A)/Å	D-H-A/°
**C15**	H15	O1[1]	0.93	2.55	3.363(3)	146.7

[1] 1-x, 1-y, 1-z

Table S14. Torsion Angles for compound **5**.

A	B	C	D	Angle/˚	A	B	C	D	Angle/˚
**Cl3**	C2	C3	C4	-179.5(2)	C7	N1	C14	C13	0.0(2)
**N1**	C1	C2	Cl3	4.8(3)	C7	C8	C9	C10	0.5(5)
**N1**	C1	C2	C3	-175.9(2)	C7	C12	C13	C14	0.5(2)
**N1**	C1	C6	Cl2	-6.6(3)	C7	C12	C13	C15	-175.0(2)
**N1**	C1	C6	C5	174.5(2)	C8	C7	C12	C11	-1.3(4)
**N1**	C7	C8	C9	-179.9(3)	C8	C7	C12	C13	179.1(2)
**N1**	C7	C12	C11	179.2(2)	C8	C9	C10	C11	-0.8(5)
**N1**	C7	C12	C13	-0.5(2)	C9	C10	C11	C12	0.1(5)
**C1**	N1	C7	C8	-2.0(4)	C10	C11	C12	C7	0.9(4)
**C1**	N1	C7	C12	177.5(2)	C10	C11	C12	C13	-179.6(3)
**C1**	N1	C14	O1	1.4(4)	C11	C12	C13	C14	-179.1(3)
**C1**	N1	C14	C13	-177.38(19)	C11	C12	C13	C15	5.4(4)
**C1**	C2	C3	C4	1.2(4)	C12	C7	C8	C9	0.6(4)
**C2**	C1	C6	Cl2	176.84(17)	C12	C13	C14	O1	-179.0(2)
**C2**	C1	C6	C5	-2.1(3)	C12	C13	C14	N1	-0.3(2)
**C2**	C3	C4	C5	-1.5(4)	C12	C13	C15	Se1	-1.7(4)
**C3**	C4	C5	C6	0.1(4)	C14	N1	C1	C2	101.8(3)
**C4**	C5	C6	Cl2	-177.18(19)	C14	N1	C1	C6	-74.6(3)
**C4**	C5	C6	C1	1.8(4)	C14	N1	C7	C8	-179.2(3)
**C6**	C1	C2	Cl3	-178.70(17)	C14	N1	C7	C12	0.3(3)
**C6**	C1	C2	C3	0.6(4)	C14	C13	C15	Se1	-176.67(16)
**C7**	N1	C1	C2	-75.1(3)	C15	C13	C14	O1	-2.8(4)
**C7**	N1	C1	C6	108.5(3)	C15	C13	C14	N1	176.0(2)
**C7**	N1	C14	O1	178.8(2)	C16	Se1	C15	C13	178.5(2)

Table S15. Hydrogen Atom Coordinates (Å×104) and Isotropic Displacement Parameters (Å2×103) for compound **5**.

Atom	*x*	*y*	*z*	U(eq)
**H3**	5845	545	2507	70
**H4**	5763	1386	1116	68
**H5**	4141	2827	906	62
**H8**	567	1108	3522	72
**H9**	-1635	710	4424	85
**H10**	-2349	1769	5615	80
**H11**	-878	3240	5977	65
**H15**	3057	5133	5471	50
**H16A**	2238	6737	6601	122
**H16B**	3304	5978	7181	122
**H16C**	1757	6464	7606	122

*III.3. Biological evaluation for compound **5***

III.3.1. NCI-60 screening data for compound **5**

Growth percent (GP) is the growth of treated culture compared to the growth of untreated cells. GP between 0 and 50 means antiproliferative properties and between -100 and 0 stands for cytotoxic properties GP (%).

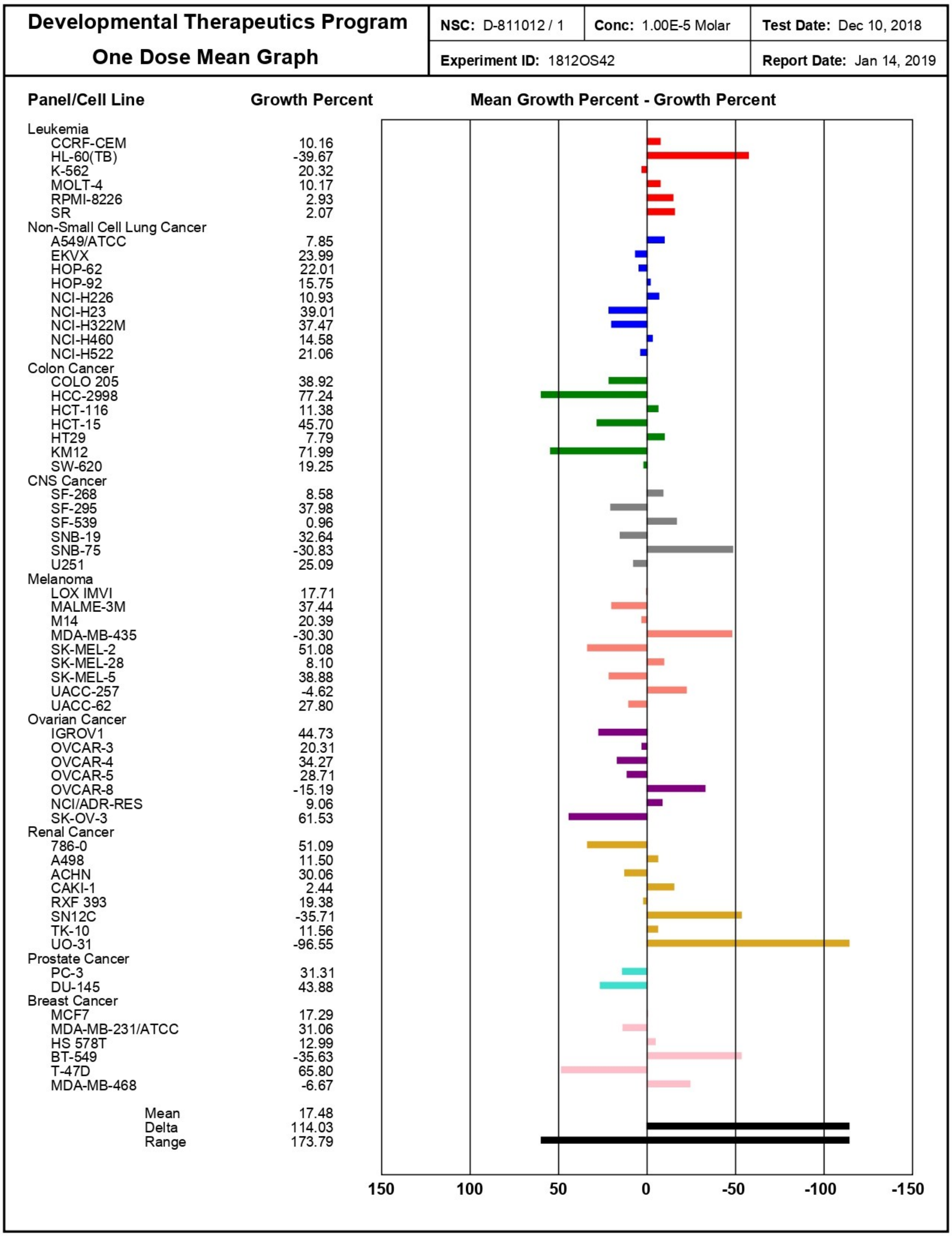

Figure S1. NCI-60 chart data for compound **5**.

IV. Spectroscopic characterization for compound 3 and 5

*IV.1. 1-(2,6-dichlorophenyl)indolin-2-one (**3**)*

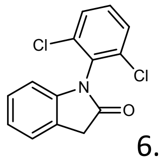

A yellow powder was obtained. Overall yield 49 %; m.p.: 120 - 122 °C; ^{1}H-NMR (400 MHz, $CDCl_3$) δ 7.50 (d, 2H, *J* = 8.4 Hz), 7.40 – 7.36 (m, 1H), 7.36 – 7.32 (m, 1H), 7.20 (td, 1H, *J* = 7.7 and 0.8 Hz), 7.09 (td, 1H, *J* = 7.6 and 0.9 Hz), 6.40 (d, 1H, *J* = 7.8 Hz), 3.77 (s, 2H). ^{13}C-NMR (100 MHz, $CDCl_3$) δ 173.62, 143.33, 135.53, 130.80, 130.48, 129.05, 127.94, 124.83, 124.31, 123.07, 109.15, 35.74 [12].

*IV.2. ((E)-1-(2,6-dichlorophenyl)-3-((methylselanyl)methylene)indolin-2-one (**5**)*

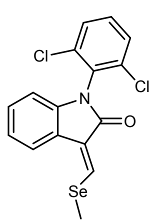

An orange powder was obtained. Overall yield 10 %; purity 95.2 %; m.p.: 173 - 174 °C; ^{1}H-NMR (600 MHz, $CDCl_3$) δ 8.46 (s, 1H), 7.59 (d, 1H, *J* = 7.3 Hz), 7.50 (d, 2H, *J* = 8.2 Hz), 7.39 – 7.34 (m, 1H), 7.21 (td, 1H, *J* = 7.7 and 1.1 Hz), 7.15 (td, 1H, *J* = 7.6 and 0.9 Hz), 6.42 (d, 1H, *J* = 7.7 Hz), 2.57 (s, 3H). ^{13}C-NMR (150 MHz, $CDCl_3$) δ 164.07, 142.00, 141.18, 136.01, 130.75, 129.11, 128.50, 125.59, 123.71, 123.20, 122.98, 109.07, 10.25. ^{77}Se-NMR (76 MHz, CDCl3) δ 245 ppm.

IV.3. NMR spectra and quantitative NMR (qNMR)

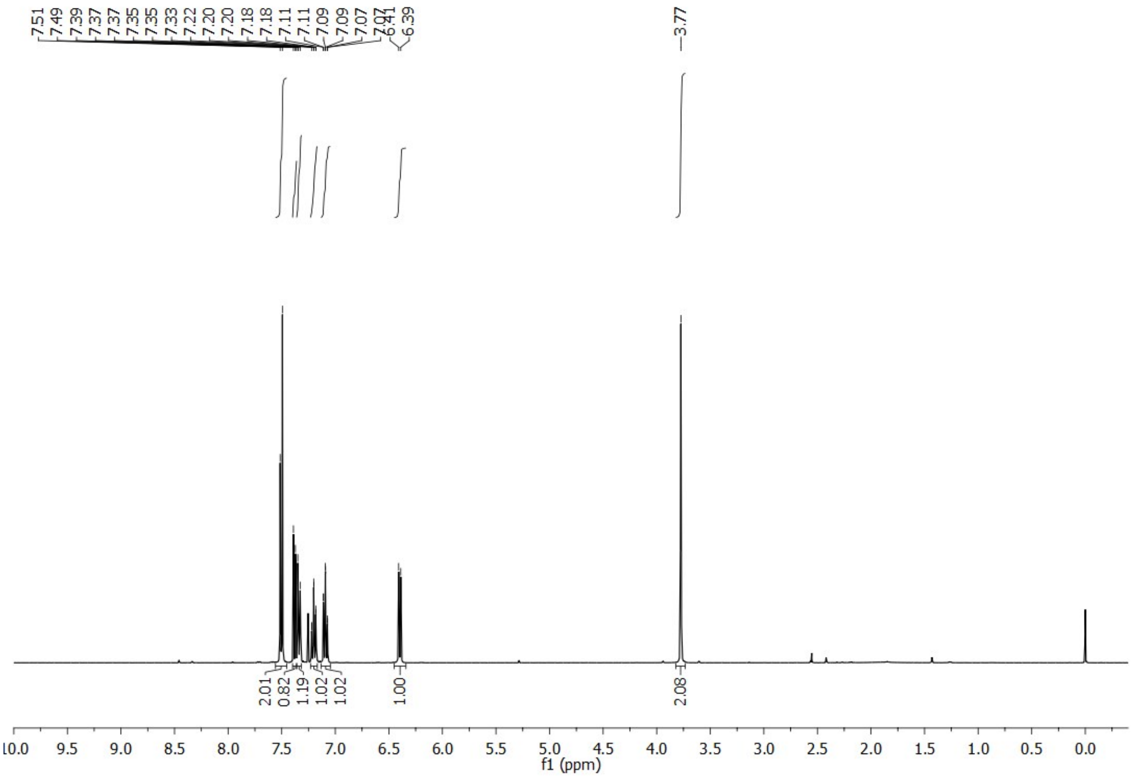

Figure S2. ^{1}H-NMR spectrum for 1-(2,6-dichlorophenyl)indolin-2-one (**3**).

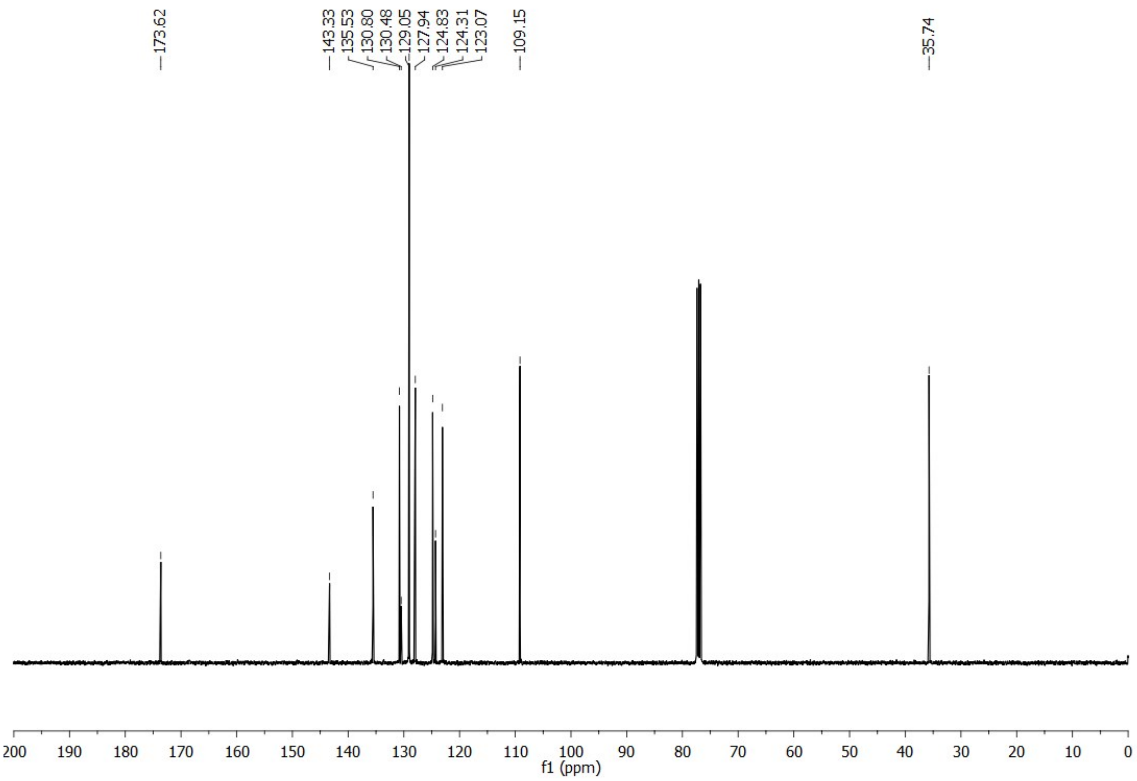

Figure S3. ^{13}C-NMR spectrum for 1-(2,6-dichlorophenyl)indolin-2-one (**3**).

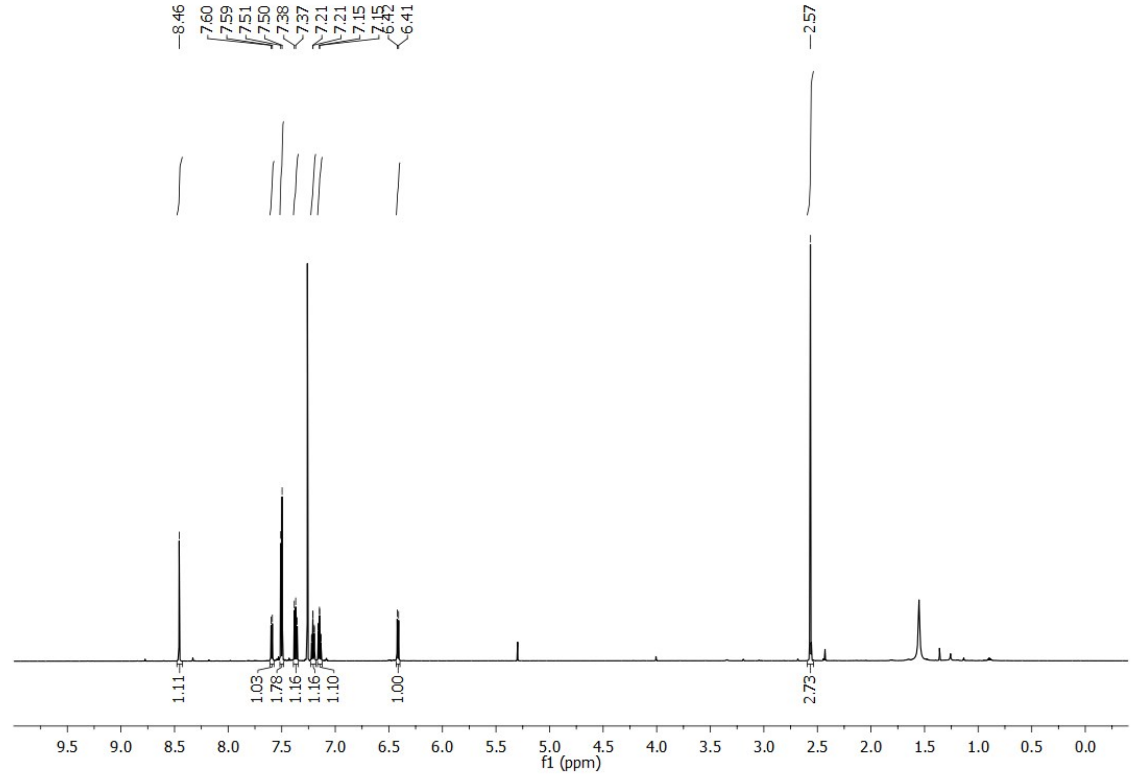

Figure S4. ^{1}H-NMR spectrum for ((*E*)-1-(2,6-dichlorophenyl)-3-((methylselanyl)methylene)indolin-2-one (**5**).

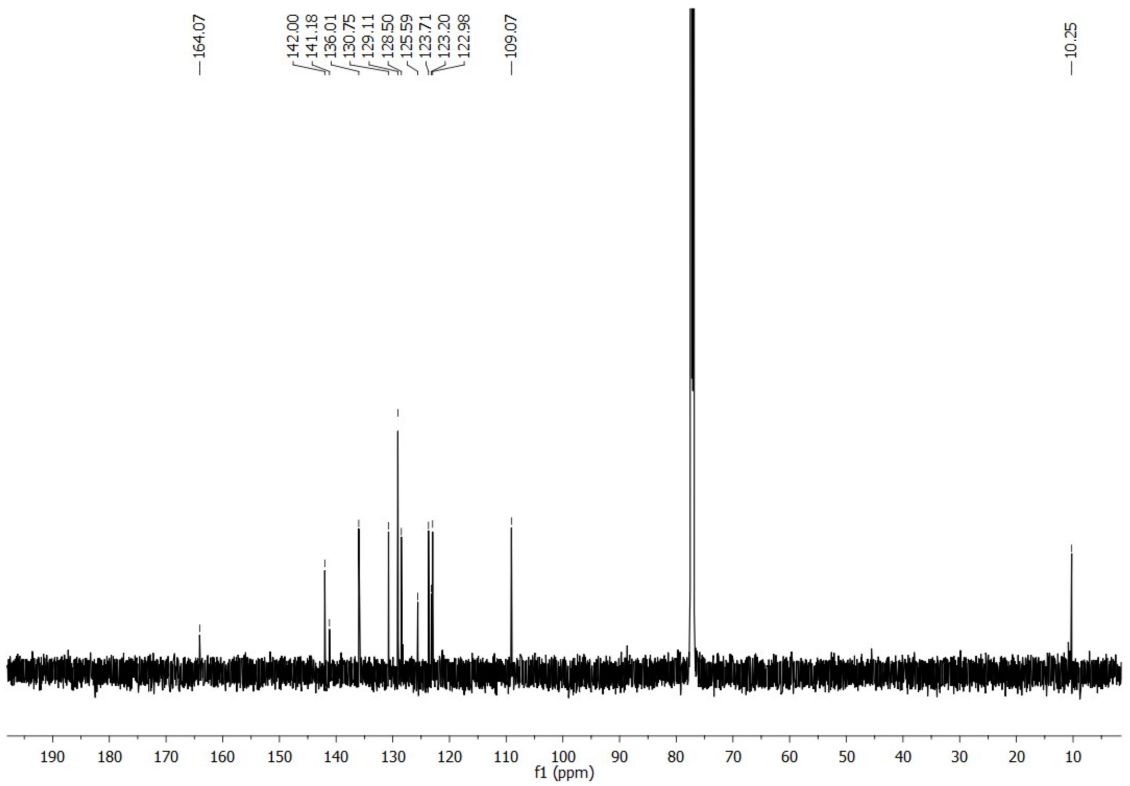

Figure S5. ^{13}C-NMR spectrum for ((*E*)-1-(2,6-dichlorophenyl)-3-((methylselanyl)methylene)indolin-2-one (**5**).

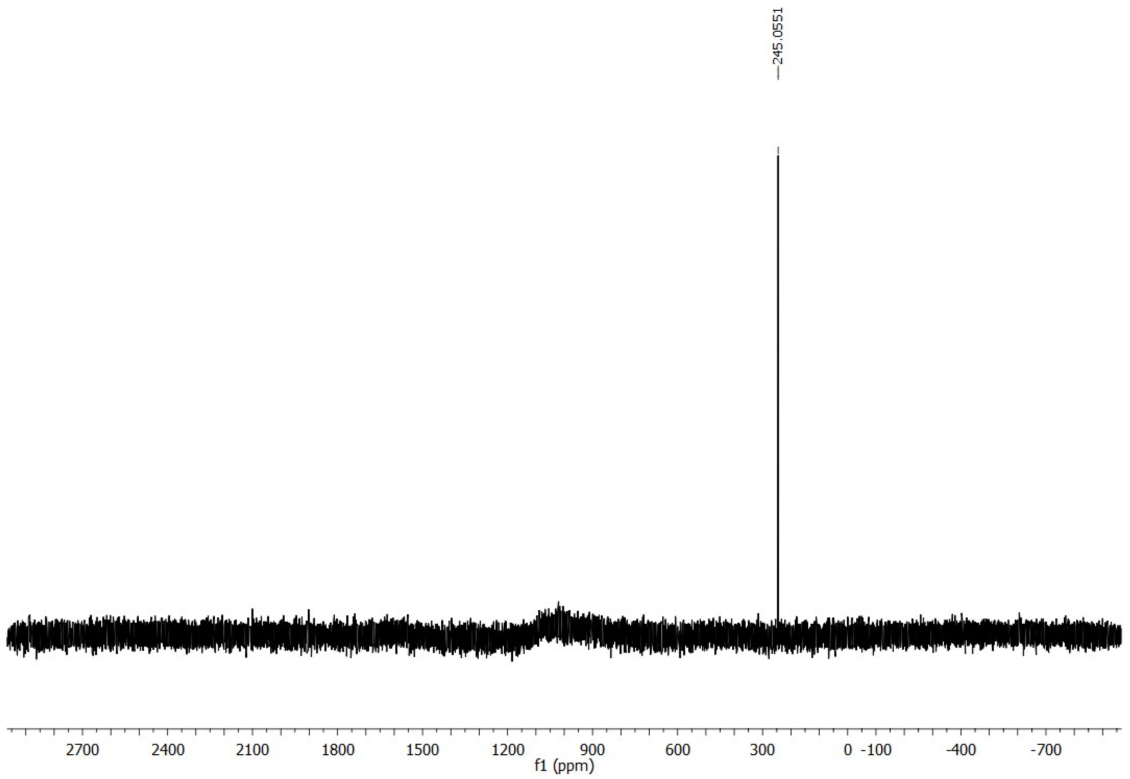

Figure S6. ^{77}Se-NMR spectrum for ((*E*)-1-(2,6-dichlorophenyl)-3-((methylselanyl)methylene)indolin-2-one (**5**).

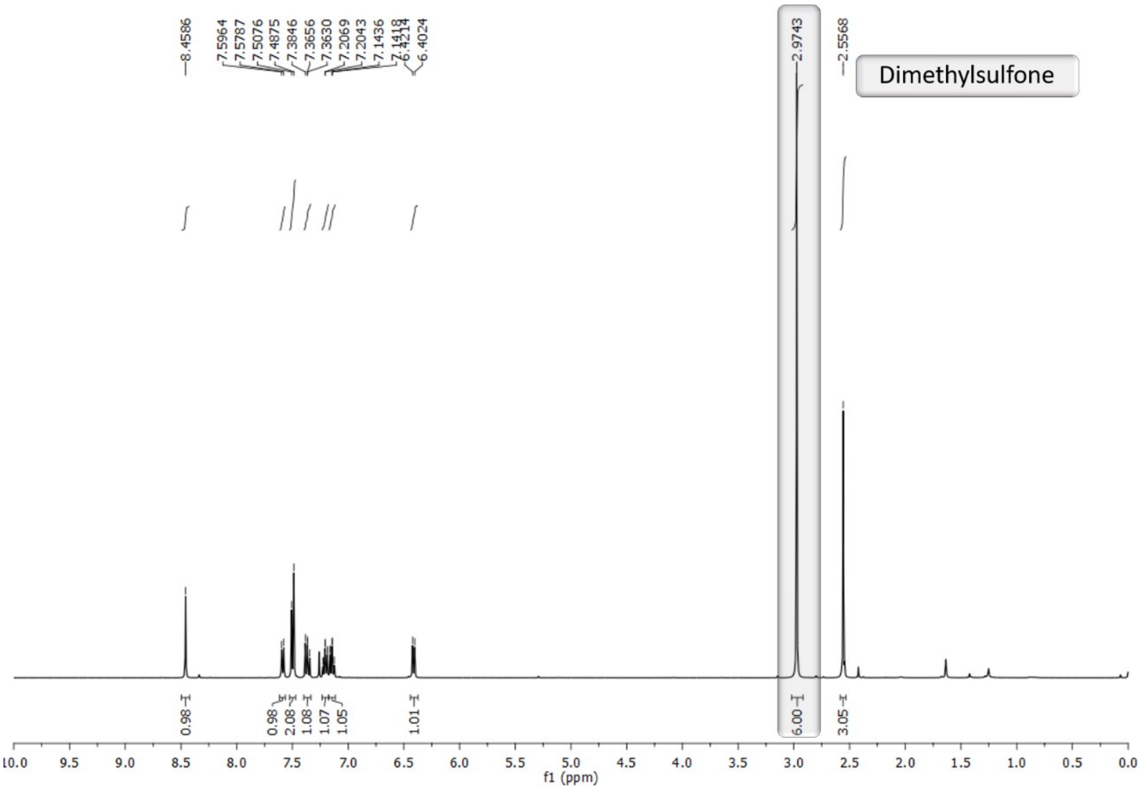

Figure S7. ^{1} H-NMR spectrum for compound **5** and dimethyl sulfone (qNMR).

V. ^{1}H-NMR spectra for reaction in the synthesis of 2-(2-((2,6-dichlorophenyl)amino)phenyl)acetyl chloride (2).

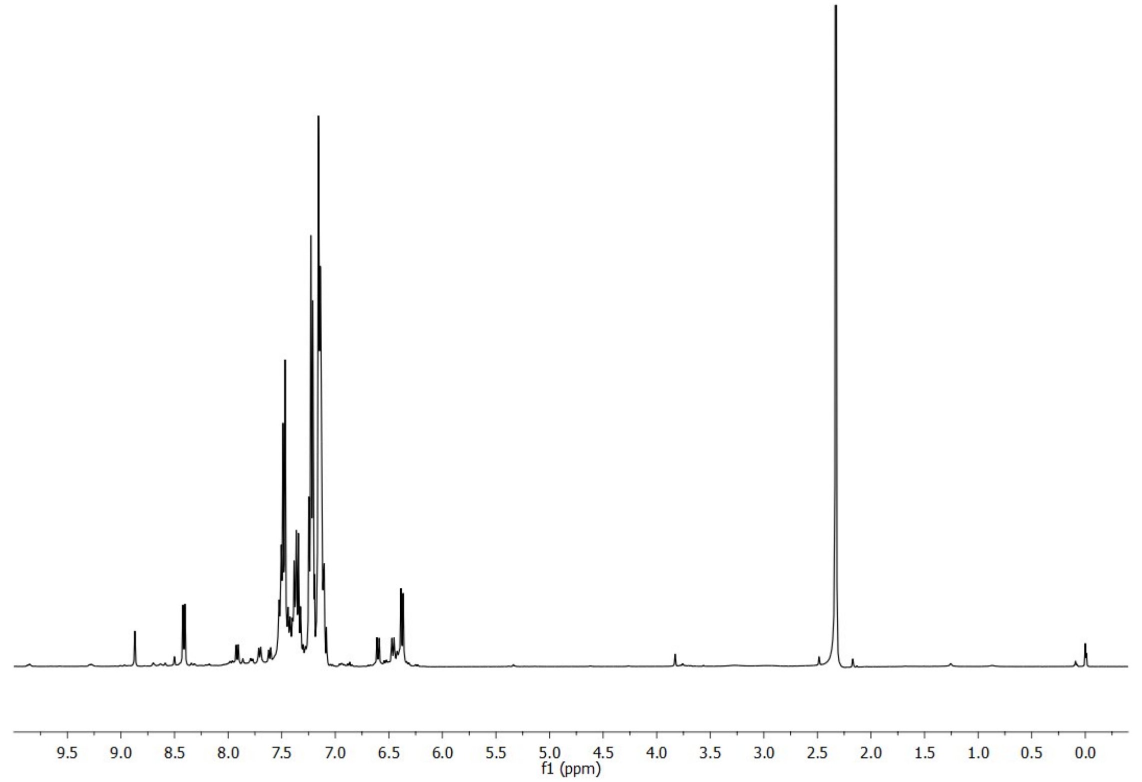

Figure S8. ^{1}H-NMR spectrum of reaction in the synthesis of 2-(2-((2,6-dichlorophenyl)amino)phenyl)acetyl chloride (2) with thionyl chloride.

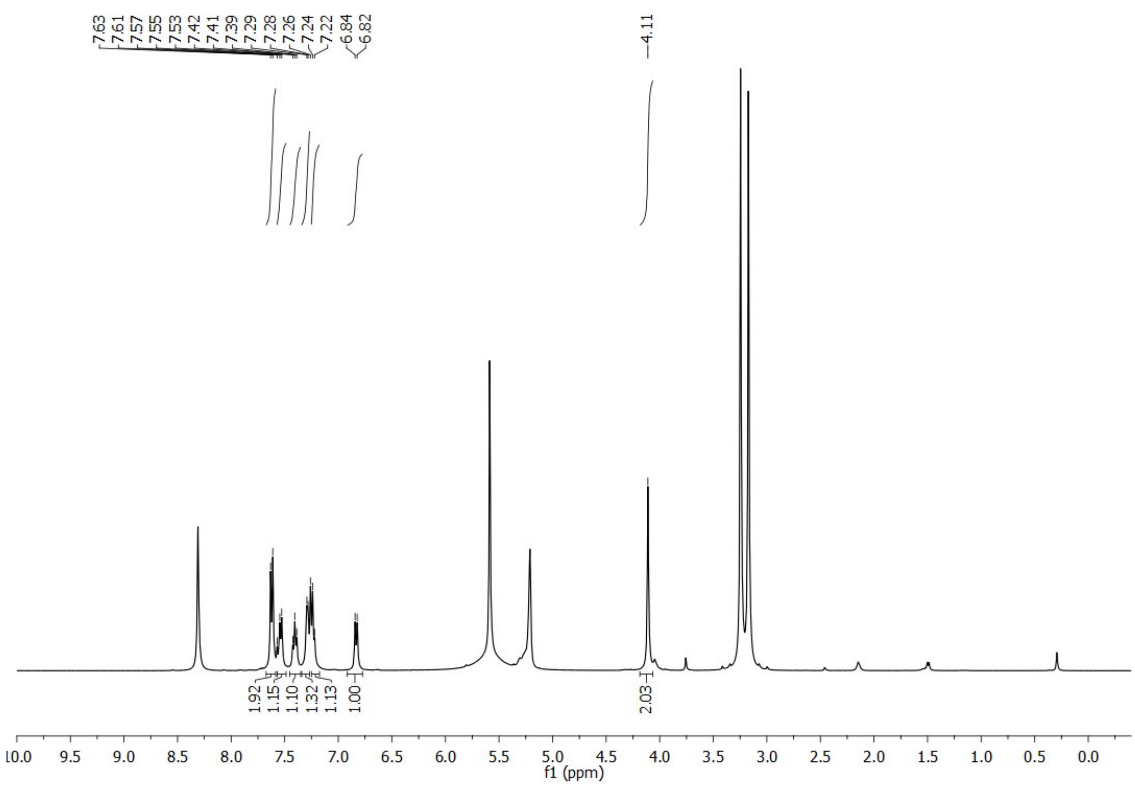

Figure S9. ^{1}H-NMR spectrum of reaction in the synthesis of 2-(2-((2,6-dichlorophenyl)amino)phenyl)acetyl chloride (2) with oxalyl chloride.

VI. ^{77}Se-NMR spectra for reaction crudes in the synthesis of alkali metal salts of hydroselenide

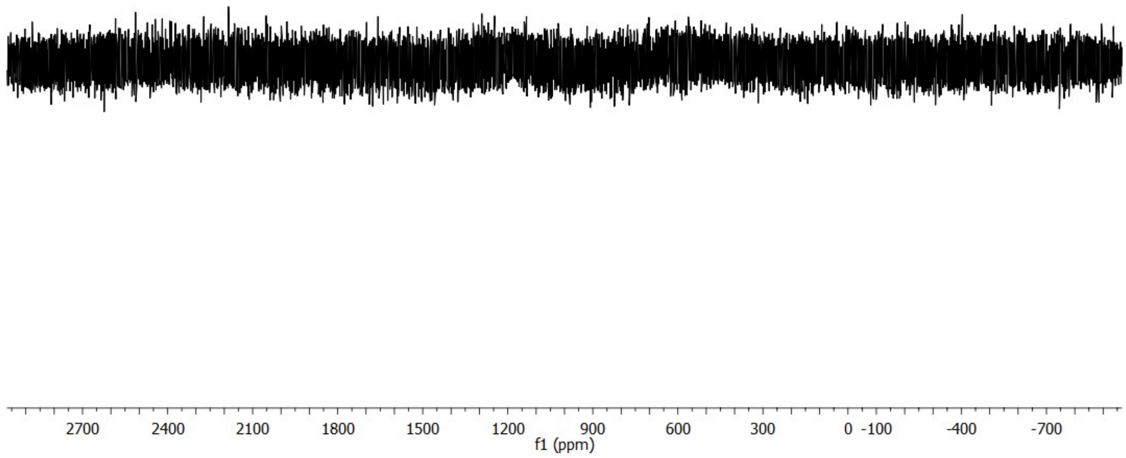

Figure S10. ^{77}Se-NMR spectrum of reaction crude in the synthesis of lithium hydroselenide with $AlLiH_4$.

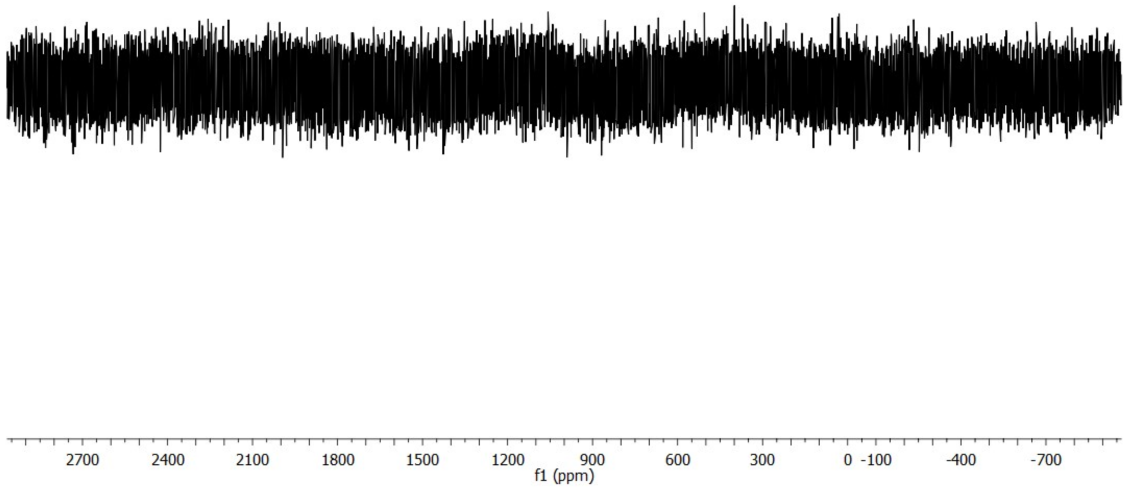

Figure S11. ^{77}Se-NMR spectrum of reaction crude in the synthesis of lithium hydroselenide with $LiEt_3BH$.

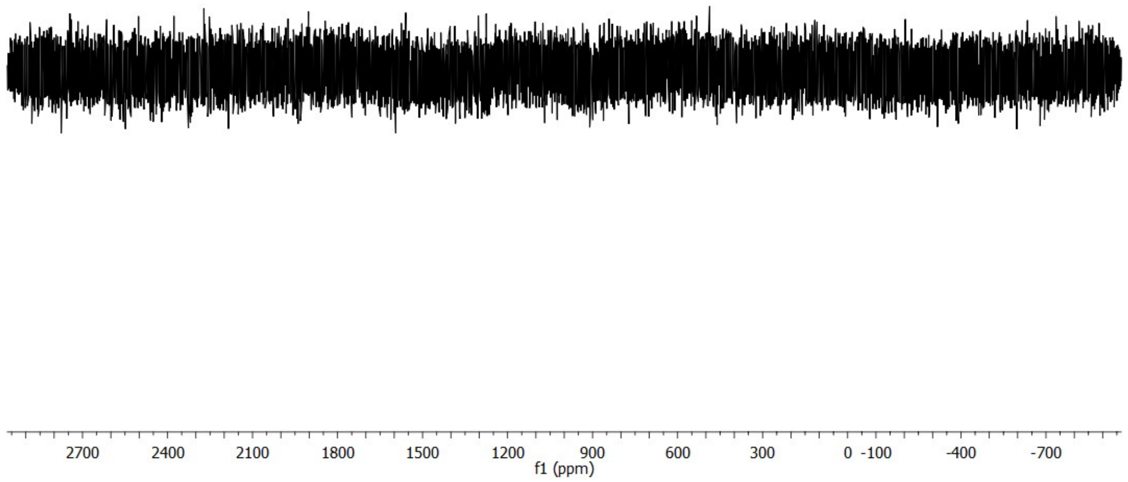

Figure S12. ^{77}Se-NMR spectrum of reaction crude in the synthesis of lithium hydroselenide with $LiAlH(OtBu)_3$.

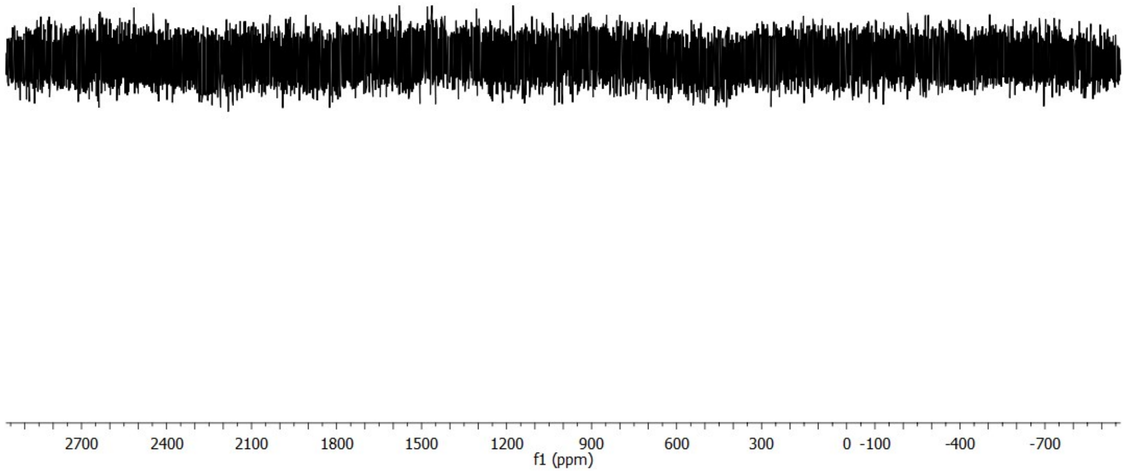

Figure S13. ^{77}Se-NMR spectrum of reaction crude in the synthesis of sodium hydroselenide with $NaBH_3CN$.

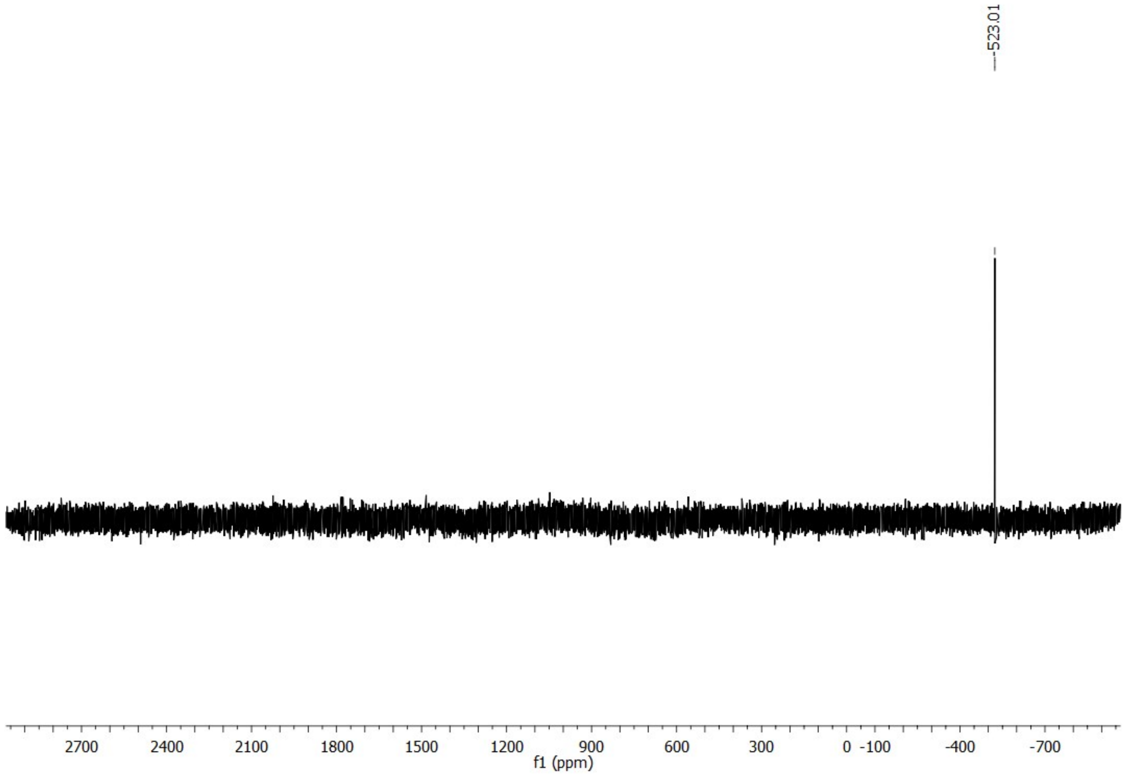

Figure S14. ^{77}Se-NMR spectrum of reaction crude in the synthesis of sodium hydroselenide with $NaBH_4$.

8. ^{1}H-NMR spectra with dimethylsulfone for quantification of crude mixture

*8.1. 1-(2,6-dichlorophenyl)indolin-2-one (**3**)*

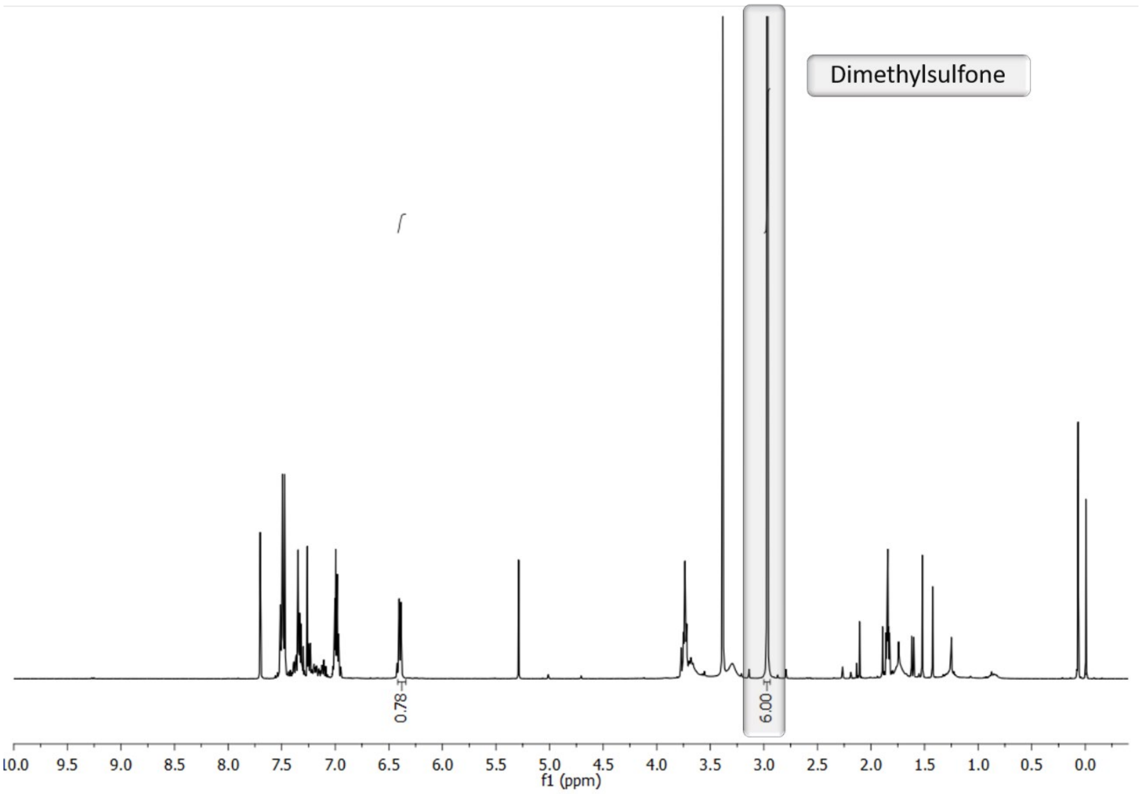

Figure S15. ^{1}H-NMR spectrum for reaction crude with $AlLiH_4$.

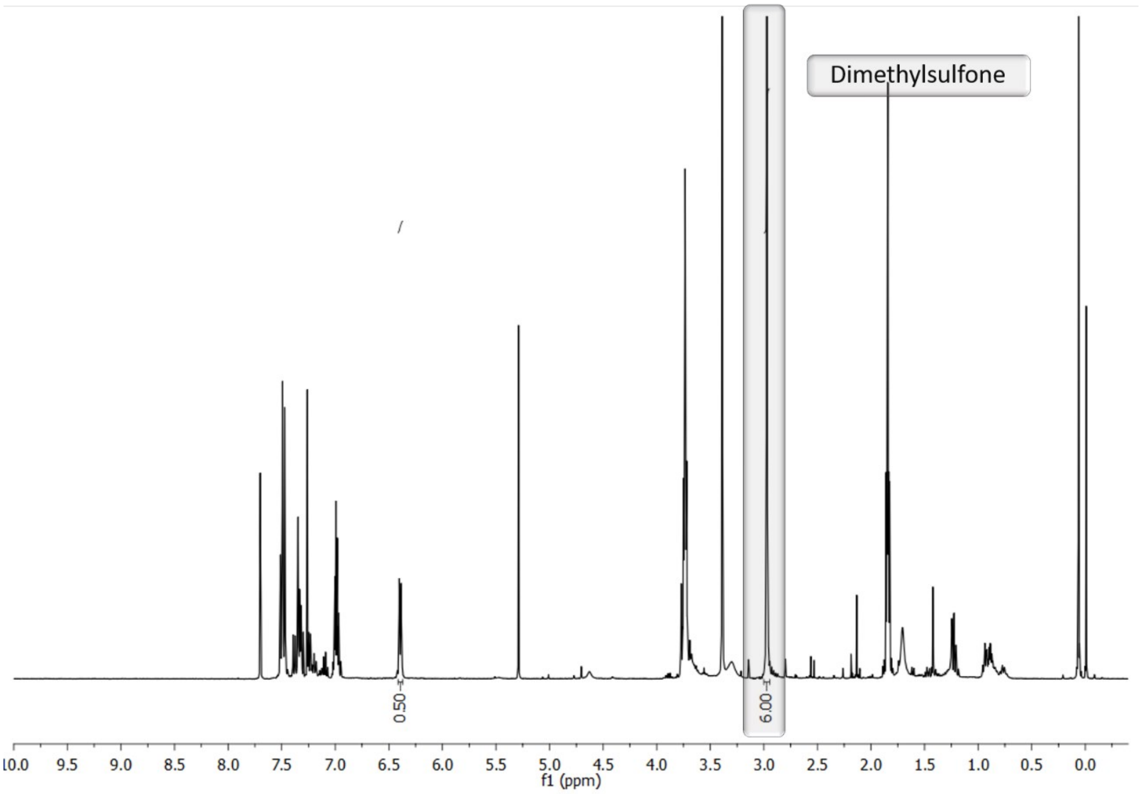

Figure S16. ^{1}H-NMR spectrum for reaction crude with $LiEt_3BH$.

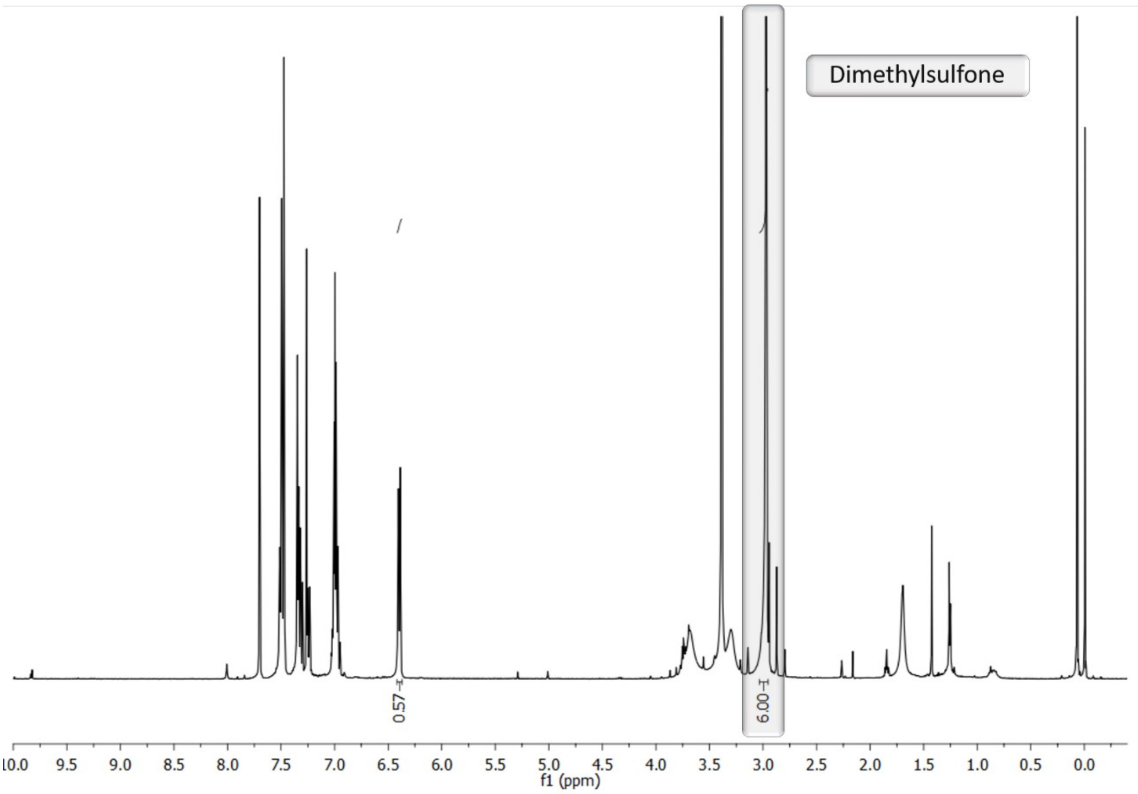

Figure S17. ^{1}H-NMR spectrum for reaction crude with $LiAlH(OtBu)_3$.

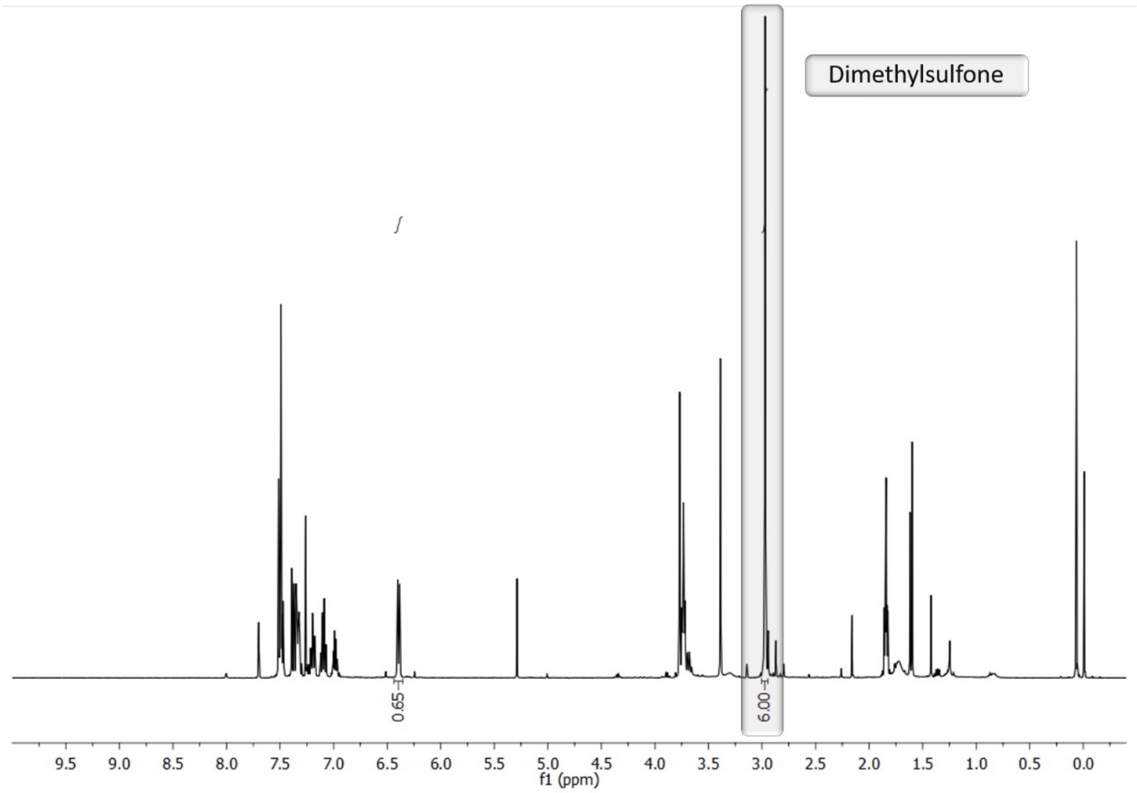

Figure S18. ^{1}H-NMR spectrum for reaction crude with $NaBH_3CN$.

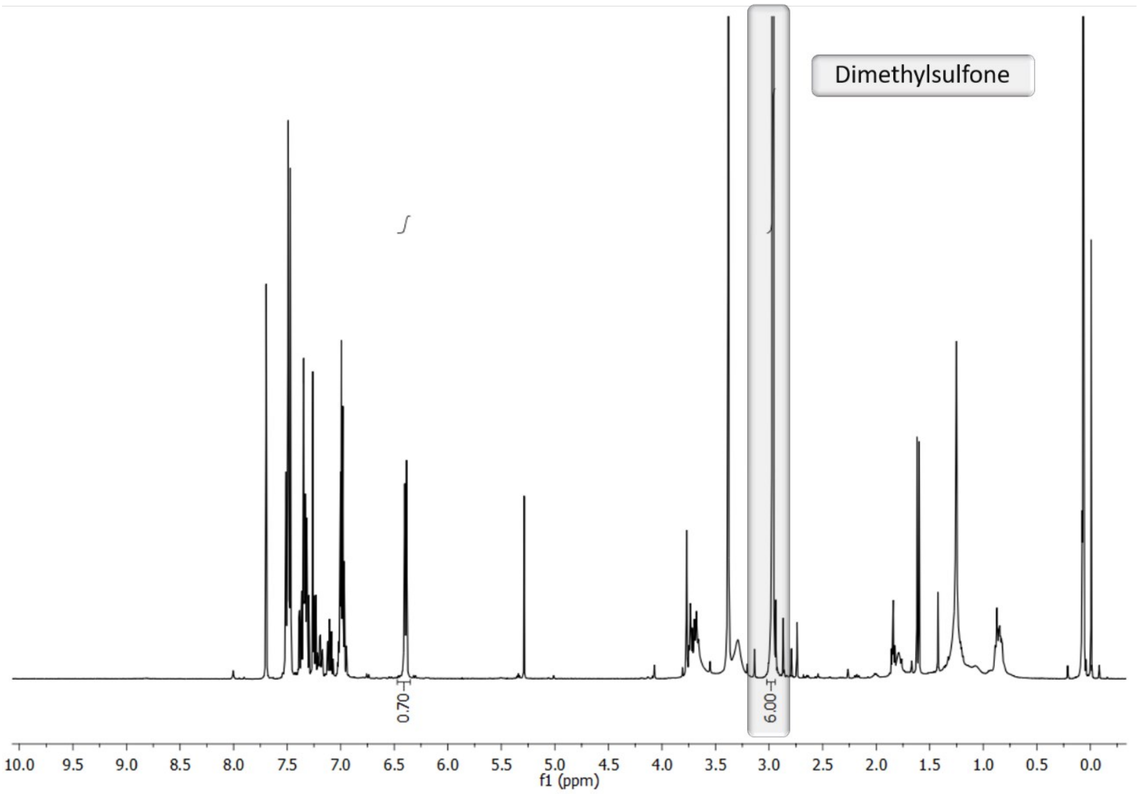

Figure S19. ^{1}H-NMR spectrum for reaction crude with $NaBH_4$.

8.2. Optimization of cyclization by N, N- dimethylformamide

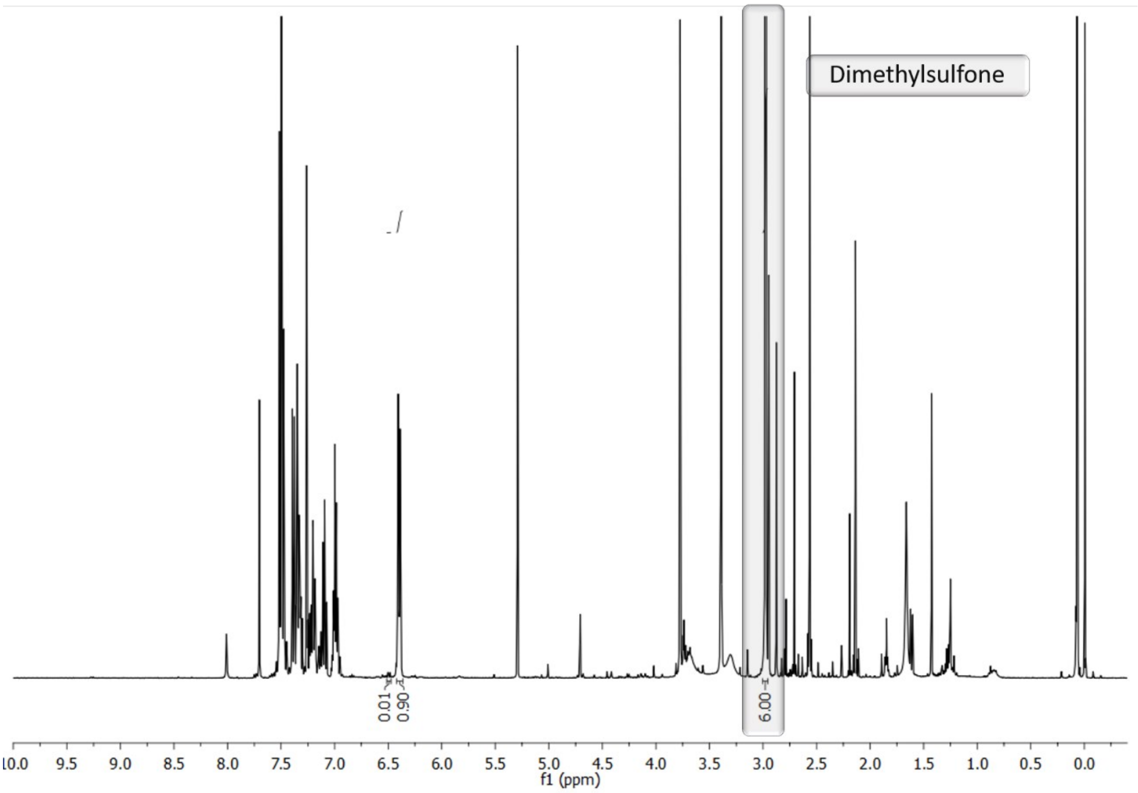

Figure S20. ^{1}H-NMR spectrum for reaction crude with 1.5 eq of *N, N-* dimethylformamide.

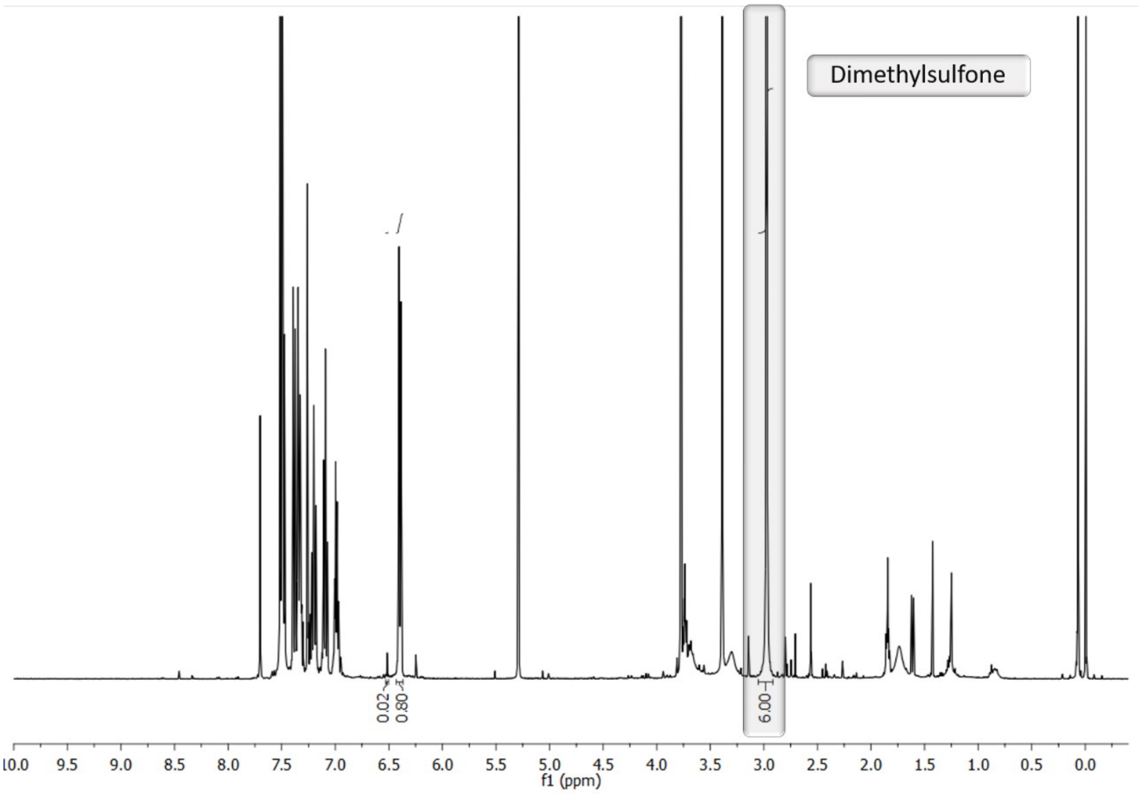

Figure S21. ^{1}H-NMR spectrum for reaction crude with 1.0 eq of *N, N*- dimethylformamide.

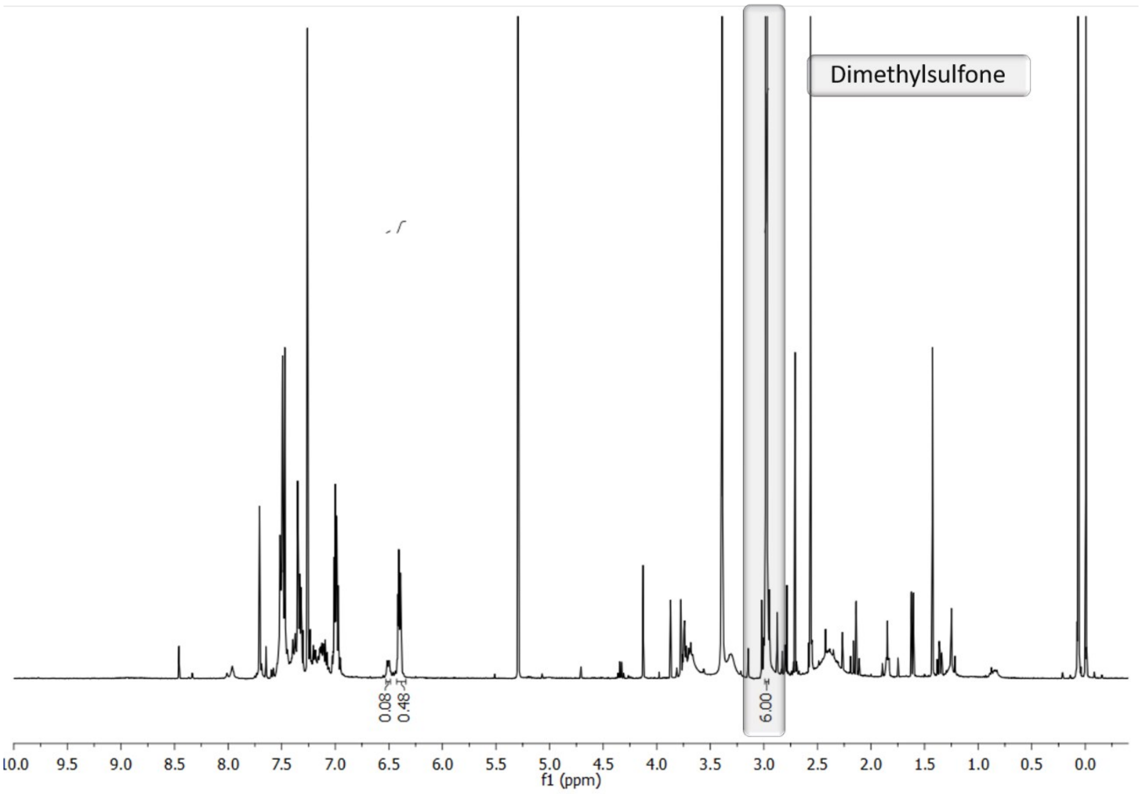

Figure S22. ^{1}H-NMR spectrum for reaction crude with 0.5 eq of *N, N*- dimethylformamide.

REFERENCES

1. M. Hanif, M. Rafiq, M. Saleem, G. Qadeer and W. Y. Wong, *Acta Crystallogr Sect E Struct Rep Online*, 2009, **65**, o583.
2. R. Cassano, S. Trombino, T. Ferrarelli, E. Barone, V. Arena, C. Mancuso and N. Picci, *Biomacromolecules*, 2010, **11**, 1716-1720.
3. D. Plano, D. N. Karelia, M. K. Pandey, J. E. Spallholz, S. Amin and A. K. Sharma, *J Med Chem*, 2016, **59**, 1946-1959.
4. M. Cushman, G. I. Georg, U. Holzgrabe and S. Wang, *J Med Chem*, 2014, **57**, 9219.
5. G. F. Pauli, S. N. Chen, C. Simmler, D. C. Lankin, T. Gödecke, B. U. Jaki, J. B. Friesen, J. B. McAlpine and J. G. Napolitano, *J Med Chem*, 2014, **57**, 9220-9231.
6. O. V. Dolomanov, L. J. Bourhis, R. J. Gildea, J. A. K. Howard and H. Puschmann, *J. Appl. Crystallogr*, 2009, **42**, 339-341.
7. G. Sheldrick, *Acta Cryst. A* 2008, **64**, 112-122.
8. R. H. Shoemaker, *Nature reviews. Cancer*, 2006, **6**, 813-823.
9. S. L. Holbeck, J. M. Collins and J. H. Doroshow, *Mol Cancer Ther*, 2010, **9**, 1451-1460.
10. K. D. Paull, R. H. Shoemaker, L. Hodes, A. Monks, D. A. Scudiero, L. Rubinstein, J. Plowman and M. R. Boyd, *J Natl Cancer I*, 1989, **81**, 1088-1092.
11. W. C. Reinhold, S. Varma, F. Sousa, M. Sunshine, O. D. Abaan, S. R. Davis, S. W. Reinhold, K. W. Kohn, J. Morris, P. S. Meltzer, J. H. Doroshow and Y. Pommier, *PLoS One*, 2014, **9**, e101670.
12. M. C. Chung, J. L. dos Santos, E. V. Oliveira, L. Blau, R. F. Menegon and R. G. Peccinini, *Molecules (Basel, Switzerland)*, 2009, **14**, 3187-3197.
